# Patient-matched tumours, plasma, and cell lines reveal tumour microenvironment- and cell culture-specific microRNAs

**DOI:** 10.1242/bio.060483

**Published:** 2024-12-23

**Authors:** Latasha Ludwig, Emma N. Vanderboon, Heather Treleaven, R. Darren Wood, Courtney R. Schott, Geoffrey A. Wood

**Affiliations:** Department of Pathobiology, University of Guelph, Guelph N1G 2W1, Canada

**Keywords:** MicroRNAs, Matched samples, Microenvironment, Cell culture, Osteosarcoma

## Abstract

MicroRNAs (miRNAs) are small non-coding RNA molecules that are present in all cell types and bodily fluids and are commonly dysregulated in cancer. miRNAs in cancer have been studied by measuring levels in cell lines, tumour tissues, and in circulation; however, no study has specifically investigated miRNA expression in patient-matched samples across all three sample types. Canine osteosarcoma is a well-established spontaneously occurring model of human osteosarcoma for which matched samples are available. We analysed a panel of miRNAs by real-time quantitative PCR and compared across patients and sample types. While some miRNAs are highly expressed in all three sample types, tumour tissue and cell lines had the most in common. There were several miRNAs that were highly expressed in plasma and tumour tissue but not in cell lines and likely represent miRNAs produced in the tumour microenvironment. Two highly expressed miRNAs were exclusive to plasma and are known to be expressed in circulating cells. This study highlights the importance of considering sample type when studying miRNAs in cancer and demonstrates the power of using patient-matched samples.

## Teaser

**Summary:** MicroRNA expression patterns across patient-matched primary canine osteosarcoma tissues, plasma samples, and derived cell lines, demonstrate differential expression across sample types within the same patient.

## INTRODUCTION

MicroRNAs (miRNA) are short, non-coding RNA molecules that regulate messenger RNA (mRNA) and are highly conserved across species. Certain miRNAs are highly expressed in specific cells, such as miR-451a in erythrocytes and miR-122 in hepatocytes, whereas others exhibit high expression across most cell types, such as miR-16 (Hu et al., 2012; Keller et al., 2022; Pritchard et al., 2012). miRNAs are also present in nearly every biological fluid, including plasma, serum, cerebrospinal fluid, and urine (O'Brien et al., 2018). As these molecules regulate mRNA, they are important in most biological processes and are dysregulated in several diseases, including cancer (Condrat et al., 2020; Peng and Croce, 2016; Sayed and Abdellatif, 2011; Van Roosbroeck and Calin, 2017). In multiple tumour types, changes in miRNA levels influence several hallmarks of cancer, including cellular proliferation, invasion, and angiogenesis (Peng and Croce, 2016; Sahabi et al., 2018; Van Roosbroeck and Calin, 2017). They can act as tumour suppressors or oncogenes (oncomiRs) and their function can be tumour-type dependent since a single miRNA can regulate multiple mRNA targets (Peng and Croce, 2016). These characteristics make miRNAs potential cancer biomarkers as well as candidate therapeutic targets. miRNAs in biological fluids are often contained within vesicles or bound to proteins, which greatly increases their stability compared to other larger RNA molecules (O'Brien et al., 2018). miRNAs exhibit ‘hormone-like activities’, as they can be selectively released into circulation where they act at a distance through targeted delivery (Neudecker et al., 2017; O'Brien et al., 2018).

The diagnostic and prognostic utility of miRNAs in biological fluids have been demonstrated in many studies (Matsuzaki and Ochiya, 2017). However, miRNAs in tumour tissues that are differentially expressed, or of prognostic significance, are not always reflected in circulating levels (Gmerek et al., 2019; Schneider et al., 2018). Cell lines are heavily relied upon in cancer research and are a key tool for mechanistic and pre-clinical research, but *in vitro* conditions infrequently reflect the *in vivo* tumour microenvironment, including blood vessels and blood components, extracellular matrix, fibroblasts, and tumour-associated immune and inflammatory cells (Mirabelli et al., 2019). *In vitro* studies lack these non-tumour cell components and therefore lack their associated miRNAs, which would normally contribute to the tumour's overall miRNA profile and its potential effects on mRNA regulation. Because cell lines are adapted to an *in vitro* growth environment, so are their gene expression patterns. Lopes-Ramos et al. (2017) showed that lymphoblastoid cell lines and fibroblast cell lines have overexpression of cell cycle genes compared to whole blood and skin. Hassan et al. (2020) found allelic loss in a canine thyroid carcinoma cell line compared to the primary tumour from which it was derived, which is reported to be common in cell lines derived from patient tumours. Kuosmanen et al. (2017) specifically demonstrated that there are miRNA profile differences between cultured and tissue-derived endothelial cells. To the authors' knowledge, comparisons of the miRNA profiles of patient-matched plasma, tumour tissue, and primary cell lines derived from these tumours, do not exist in the literature.

Osteosarcoma (OS) is the most common malignancy of bone in both humans and dogs; the incidence is much higher in dogs, and it has an even more aggressive clinical behaviour (Simpson et al., 2017). Since OS is a rare cancer type in humans, canine OS is considered an important and highly relevant model for the human disease (Beck et al., 2022; Van Der Weyden et al., 2020). miRNAs are dysregulated in both human and canine primary and metastatic OS and are key regulators of a variety of pathways involved in tumour cell proliferation and invasion, as well as establishment of metastatic disease (Leonardi et al., 2021; Sahabi et al., 2018). Previous studies have investigated and reported on miRNA in canine samples including tissue, cell lines and plasma from osteosarcoma patients (Fenger et al., 2016; Gourbault and Llobat, 2020; Leonardi et al., 2021). The focus of the current study was to use matched samples from the same patient to investigate how sample type influences measurement of miRNA. We sought to compare miRNA levels in tumour tissue, cell lines derived from that tissue, and plasma all from the same patient. This comparison across sample types from the same individual offers unique insights into the biological processes occurring and such patient-matched samples can be difficult to obtain, especially in rare tumour types such as OS in humans. While there are hundreds of miRNAs that have been annotated in dogs, we selected those of particular interest and value to canine osteosarcoma based on literature and those expressed in the sample types analysed. These comparisons yield insights into the fundamental biology of miRNA expression and provide new knowledge that can be applied to miRNA research and biomarker discovery.

## RESULTS

### Cell lines do not completely reflect the tissue from which they were derived

The three OS cell lines (OVC-cOSA-75, −78, and −31) had matched tissue available for comparison. Most of the miRNAs had no expression (Ct>35) in any cell line or tissue (32/59 miRNAs, 54%). After correction, the Ct values ranged from 23.66 to 35.00 in the cell lines and 22.60 to 35.00 in the tissues, with miR-125b-5p having the greatest average expression in both sample types. Nine miRNAs (15%) were expressed in the tissue samples but not in the cell lines (miR-379-5p, miR-885-5, miR-452-5p, miR-142, miR-200c-3p, miR-126-5p, miR-127-3p, miR-150-5p, miR-451a). MiR-183-5p was expressed in all cell lines but not in the tissues. Four miRNAs (6.8%) had no expression (Ct>35) across all samples (miR-326, miR-1844, miR-206, and miR-211-5p) and were removed from further analyses.

**Fig. 1.**
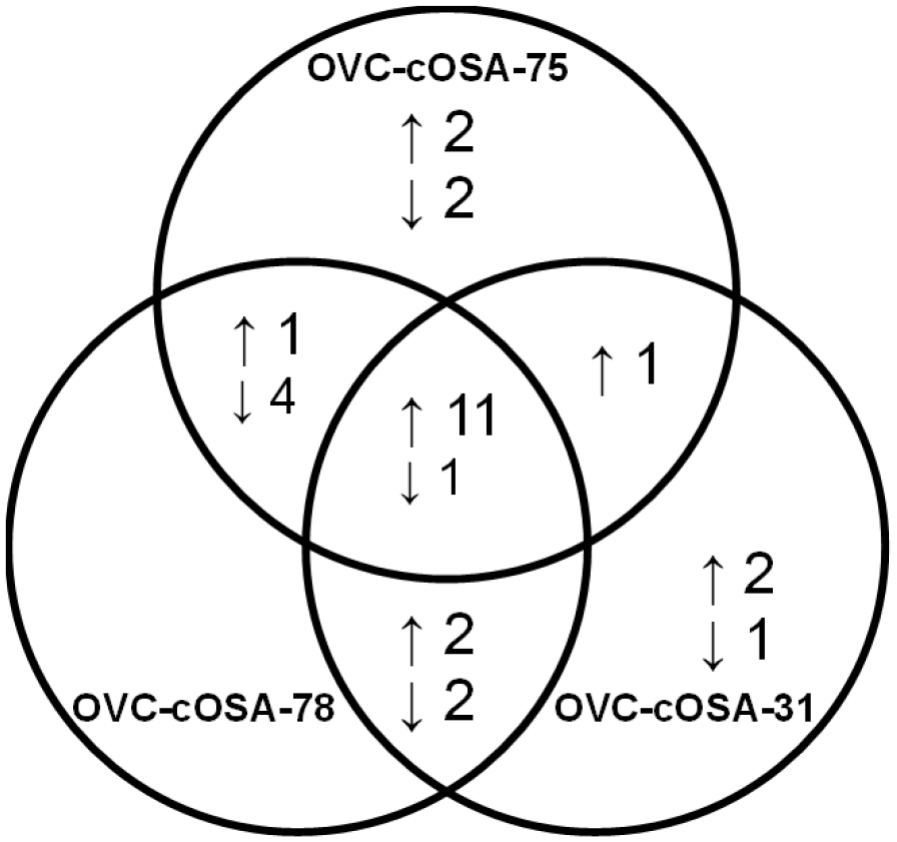
**Venn diagram of the number of upregulated and downregulated miRNAs in tissue compared to cell lines derived from three different canine osteosarcoma patients.** Differentially expressed miRNAs determined by RT-qPCR, ↑=upregulated miRNA, ↓=downregulated miRNA. Only 11 miRNAs are similarly upregulated, and one miRNA is similarly downregulated in tissues compared to the derived cell lines across all patients.

The fold-differences between tissues and cell lines for each miRNA were determined within individual patients and as a group (Table 1). As a group, 18 miRNAs (18/55 miRNAs, 33%) had higher expression in tissues compared to cell lines (fold-difference range: 2.18 to 104.59), of which five (5/18 miRNAs, 28%) were significantly different (*P*<0.05). 11 of 18 (61%) miRNAs had higher expression in all three patients when comparing the tumour tissue to its derived cell line (Fig. 1). Nine miRNAs (9/55 miRNAs, 16%) had lower expression in tissues compared to cell lines (fold-difference range: −15.78 to −2.10; *P*>0.05; Table 2). Only miR-183-5p had lower expression in tissues in all three patients (Fig. 1, centre of Venn diagram), however, the magnitude of the fold-difference in metastatic OVC-cOSA-31 (−2.31) was much less than that of the primary OS cases (−37.69 and −24.35) (Table 2). Of those with average fold-differences between tissues and cell lines, most demonstrated variability between individual samples (Fig. 1). 28 miRNAs (28/55 miRNAs, 51%) had no average significant fold-difference (fold-difference<2) between tissues and cell lines (Table S2). However, only six (6/55 miRNAs, 11%; miR-30c-5p, let-7b-5p, miR-95-3p, miR-885-5p, miR-151a-5p, and miR-185-5p) exhibited less than a 2-fold difference across all three patients (Table S2).

**Table 1.**
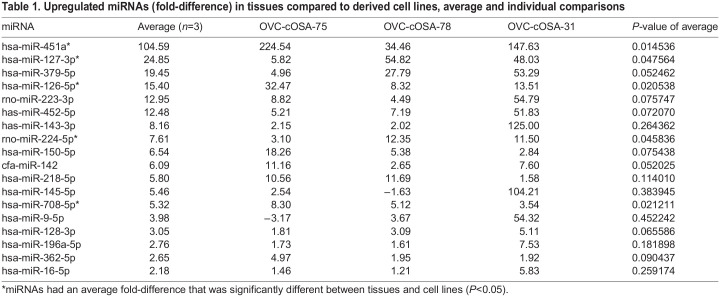
Upregulated miRNAs (fold-difference) in tissues compared to derived cell lines, average and individual comparisons

**Table 2.**
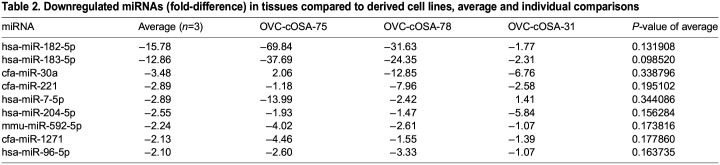
Downregulated miRNAs (fold-difference) in tissues compared to derived cell lines, average and individual comparisons

### Plasma, tissues, and cell lines express miRNAs reflective of their composition

**Fig. 2.**
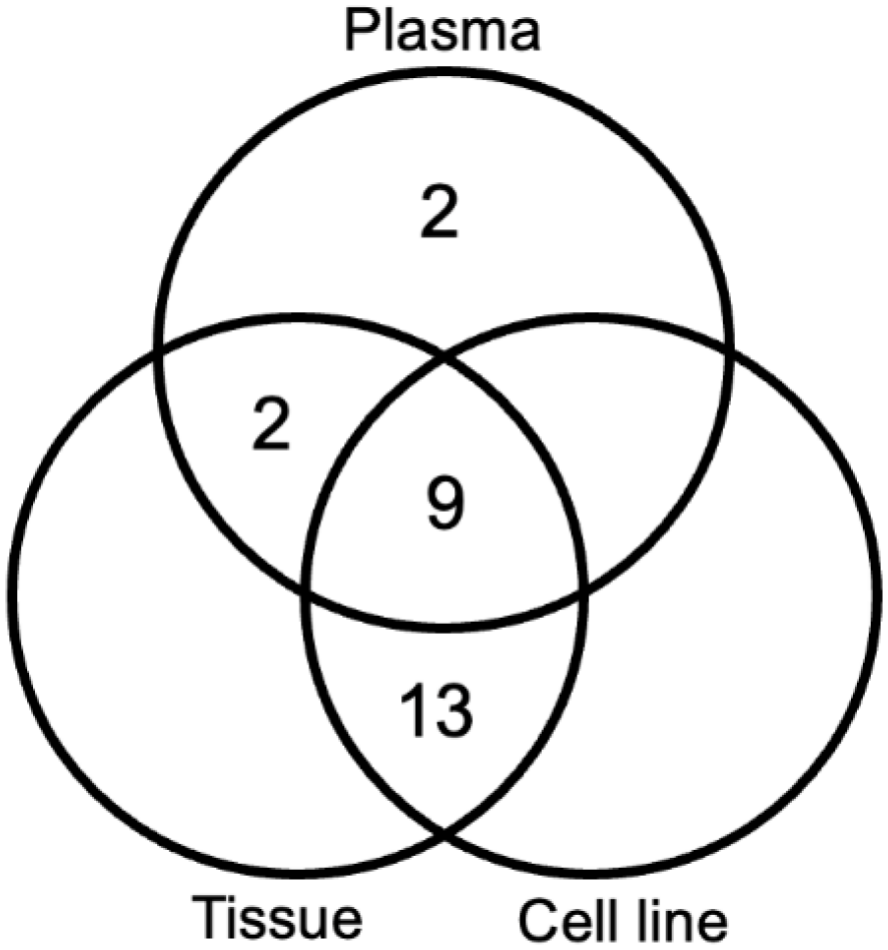
**Venn diagram of the sample types investigated (tissue, derived cell line, and plasma) by RT-qPCR, and the number of miRNAs highly expressed in these sample types.** An miRNA is considered highly expressed in multiple sample types if the cycle threshold (Ct) values are within three of the lowest Ct value. Cell lines and tumour tissue shared more highly expressed miRNAs than either shared with plasma. There were no highly expressed miRNAs unique to either tumour tissue or cell lines.

**Fig. 3.**
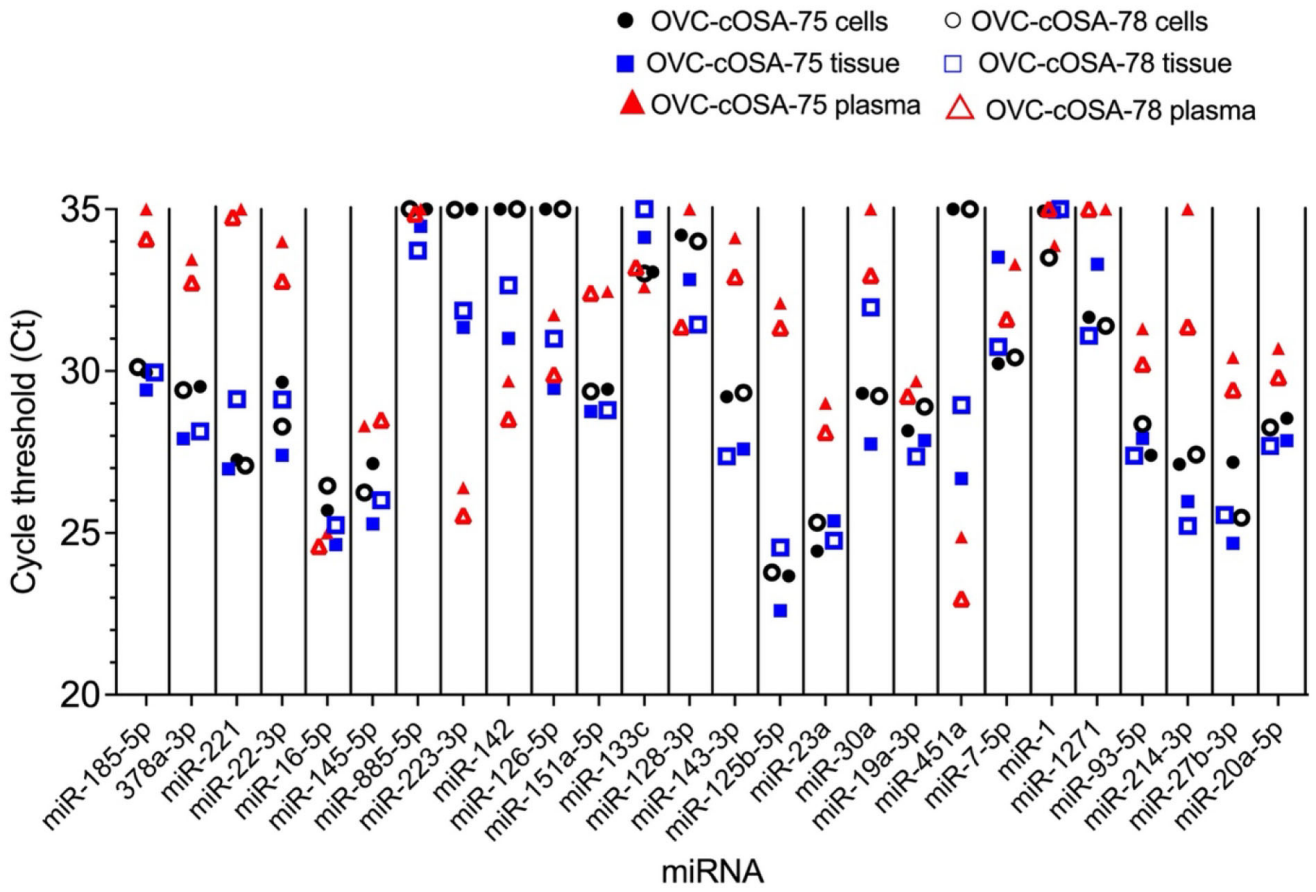
**Cycle threshold (Ct) values for miRNAs investigated in two patients (OVC-cOSA-75 and OVC-cOSA-78) with matched tissue, derived cell line, and plasma samples (all *n*=1).** miRNAs were analysed by RT-qPCR and calibrated Ct values were plotted for each miRNA and samples. Tissues generally have a close relationship with either the derived cell line or the plasma as the whole tissue is a combination of both components.

OVC-cOSA-75 and OVC-cOSA-78 had a matched derived cell line, tissue, and plasma available for comparison (Table S3). Direct fold-difference comparisons were not possible as there are multiple differences in methods, and the endogenous controls for normalization are not the same. As such, only descriptive comparisons are possible based on the calibrated Ct values. Tissue samples were more similar to their derived cell lines than their plasma (Figs 2 and 3). Nine miRNAs (9/26 miRNAs, 35%) had Ct values within a difference of three cycles for matched tissue, cell line, and plasma samples. 22 miRNAs (22/26 miRNAs, 85%) had Ct values within a difference of three cycles for tissues and cell lines, whereas only 11 (11/26 miRNAs, 42%) were similarly shared between tissues and plasma samples. No miRNAs were shared exclusively between plasma and cell lines, and additionally, plasma had two uniquely highly expressed miRNAs (miR-223 and miR-451a). Ten miRNAs (10/26 miRNAs, 39%) had a Ct value at least three cycles higher (less expression) in plasma compared to matched tumour tissues and cell lines. MiR-223-3p, miR-142, and miR-451a (3/26 miRNAs, 12%) had higher expression (lower Ct) in plasma compared to cell lines or tissues. Five miRNAs (5/26 miRNAs, 19%) had no expression (Ct=35) in one or both cell lines but some degree of expression (Ct<35) in tissue and plasma (miR-885-5p, miR-223-3p, miR-142, miR-126-5p, miR-451a).

### MiR-34a-5p expression is correlated with cell line doubling time

**Fig. 4.**
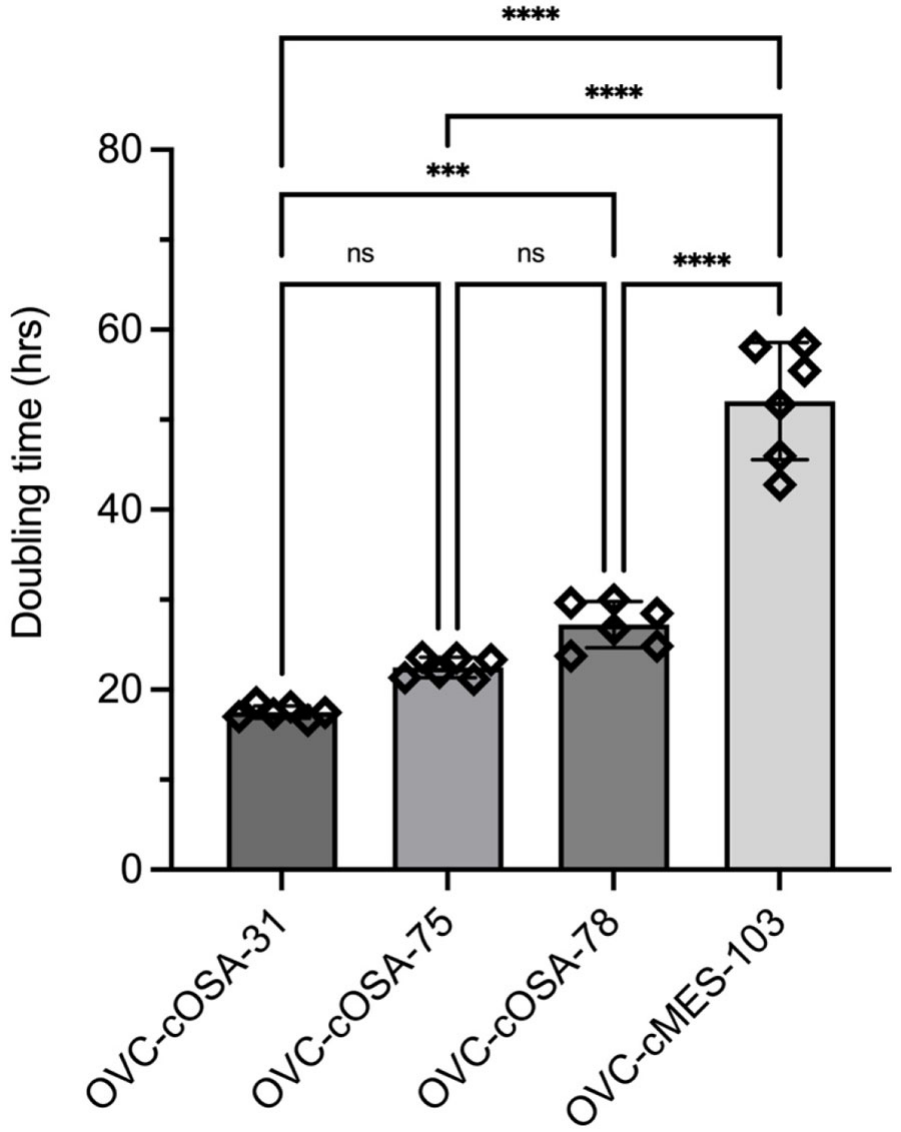
**Average cell line (six replicates per cell line) doubling time (h) showing that OVC-cMES-103 doubling time is significantly slower than all other cell lines.** OVC-cOSA-31 has a faster doubling time than OVC-cOSA-78 and OVC-cMES-103. Error bars are standard deviation. ***P<0.001, ****P<0.0001, ns, *P*>0.05.

**Fig. 5.**
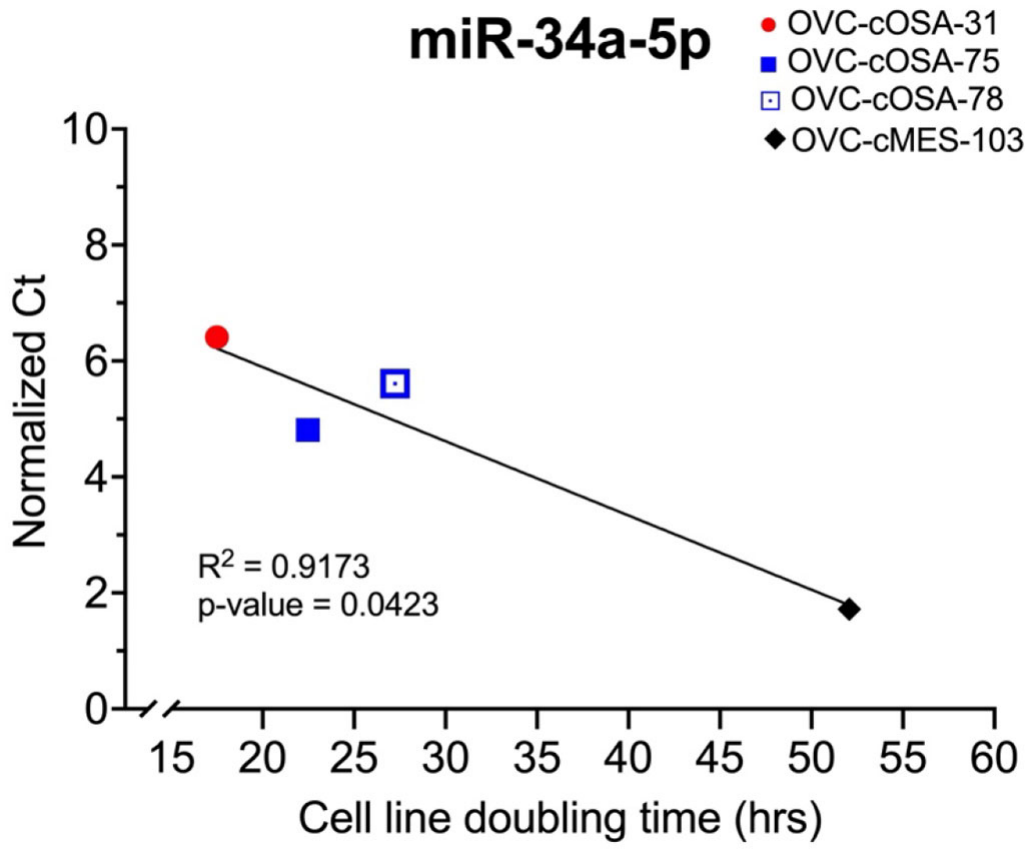
**Mir-34a-5p demonstrates a statistically significant (*P*<0.05) negative correlation (by Pearson correlation coefficient) with average cell line doubling time (h).** There is no expression of miR-34a-5p in OVC-cOSA-31 and highest expression in OVC-cMES-103 that corresponds to the fastest and slowest cell line doubling time, respectively.

Since miRNAs can influence cell proliferation rate and we observed some stark differences between the cell lines with regards to miRNA expression, it was of interest to explore whether a correlation existed between miRNA levels and cell line doubling time. OVC-cOSA-31 had the shortest average doubling time of 17.51±0.70 h [mean±standard deviation (SD)], which was significantly shorter than OVC-cOSA-75 and OVC-cOSA-78 at 22.47±1.14 h and 27.24±2.57 h, respectively (Fig. 4). OVC-cMES-103 had the longest average doubling time of 52.07±6.51 h, which was significantly different from the other cell lines (Fig. 4). The average doubling time was compared to the normalized Ct value to investigate potential statistical correlations. Only linear relationships were investigated due to the low number of data points (*n*=4). MiR-34a-5p demonstrated a statistically significant correlation with cell line doubling time, having the highest expression in OVC-cMES-103 (Ct=29.70), lower expression in OVC-cOSA-75 and −78 (Ct=32.38 and 33.79, respectively), and no expression in OVC-cOSA-31 (Ct=35.00) (Fig. 5). Using TargetScan and miRDB, MDM4, HCN3, and FAM76A were identified in both databases as genes targeted by miR-34a-5p (Table S4).

## DISCUSSION

### Using matched patient samples to further understand disease and determine appropriate therapeutic targets

To develop a greater understanding of tumour-specific miRNAs, a derived cell line in conjunction with the tumour tissue and blood samples is helpful as cell line examination alone eliminates the miRNAs coming from interstitium, immune cells, and vasculature. The miRNA profile from each of these sample types requires appropriate interpretation and should be considered in combination to achieve an accurate understanding of miRNAs on the disease of interest. Multiple studies have examined tissue samples and blood from the same patient and identified commonly dysregulated miRNAs (An et al., 2019; Geusau et al., 2020; Thakur et al., 2016). When miRNAs are similarly expressed in blood and tumour tissue this could be due to the release of the miRNAs by neoplastic cells into blood, including as circulating tumour cells, or alternatively, it might simply reflect widespread expression of the miRNAs in many cell types, such as occurs with miR-16. To understand the latter, a direct comparison of expression levels in tissue and blood from healthy patients is crucial, such as done by Thakur et al. (2016).

miRNAs can serve as therapeutic targets; however, this has proven difficult for several reasons. Although potential target miRNAs have been identified, drugs targeting miRNAs have not successfully achieved approval for clinical use and have been terminated in early clinical trials (Forterre et al., 2020). One of the unanticipated consequences of miRNA therapeutics is their action on immune cells, which in one trial resulted in patient mortality (Forterre et al., 2020). These challenges highlight the importance of understanding the specific biological processes of individual miRNAs by utilizing combined cell culture and tissue studies.

### Cell line miRNAs do not reflect those in the tumour microenvironment

Although tissues and their derived cell lines had a similar quantity of miRNAs expressed (85% of miRNAs investigated; Fig. 3), stark differences exist in miRNAs expressed by the tumour microenvironment. The circulatory system, including blood vessels and blood, is an obvious component of tissues that is absent *in vitro*. The complete lack of expression of miR-451a and miR-126-5p in cell lines, and high expression in tissues (Table 1 and Table S3), directly reflects the lack of these components in cell lines. MiR-451a is highly expressed in erythrocytes and leukocytes, and miR-126-5p is enriched in endothelial cells, as well as platelets (Chistiakov et al., 2016; Keller et al., 2022; Koenig et al., 2016; Pritchard et al., 2012). Plasma is in contact with both endothelial cells and erythrocytes, so the similar or greater levels of these two miRNAs in plasma compared to matched tissue likely reflect this close association (Fig. 3 and Table S3).

Multiple immune cell miRNAs had higher expression in tissue compared to cell lines, but this difference was not statistically significant (Table 1, Fig. 1; Table S3). These included miR-223-3p and miR-142, known to be associated with neutrophils, and miR-150-5p which is associated with lymphocytes (Pritchard et al., 2012). MiR-223-3p and miR-150-5p were not expressed in cell lines and the lack of statistical significance is likely due to the generally low proportion of neutrophils and lymphocytes compared to tumour cells in OS. However, leukocytes and/or their subcellular components can be present in plasma depending on the quality of sample acquisition and processing. Thus, the high expression of miR-223-3p and miR-142 in the plasma samples compared to their matched tissue or cell line could reflect leukocyte-associated miRNAs.

Given the differences outlined above, using cell lines as a control for whole tissue samples in miRNA studies could be problematic. The published studies examining canine OS miRNA use different controls when examining whole tissue. Some studies have normal bone, while others have used osteoblastic cell lines as their controls (Dailey et al., 2021; Fenger et al., 2016). Whole bone is composed of relatively few osteoblasts (the cell type from which OS resembles), demineralization techniques affect the quality and quantity of nucleic acids, and bone marrow components may contaminate the sample (Singh et al., 2013). Given these potential problems, the use of an osteoblastic cell line as a control is appealing. In a previous study that compared primary canine osteoblastic cell cultures to OS tissues, the two miRNAs with the greatest fold-difference were miR-451a and miR-126, which are highly enriched in erythrocytes and endothelial cells and thus reflect the lack of circulatory system-associated miRNAs in the cell lines (Fenger et al., 2016). Conversely, appropriate interpretation of miRNA profiles from whole tissue is essential (Witwer and Halushka, 2016). Although inflammatory cell-derived and other tumour microenvironment miRNAs are important in the overall biology and behaviour of a tumour, they do not reflect miRNAs produced by the tumour cells themselves. For example, in inflammatory lung disease, one study demonstrated that neutrophils transfer miR-223 to pulmonary epithelial cells which protected against acute lung injury (Neudecker et al., 2017). The extent to which this occurs in neoplasia is not established.

### Plasma is reflective of its components, including the release of tissue-related miRNAs

Accessible bodily fluids, such as plasma, can provide a convenient sample and miRNAs in plasma can serve as potential biomarkers in cancer and other diseases (Condrat et al., 2020; Matsuzaki and Ochiya, 2017; Smolarz et al., 2022). miRNAs are released into the fluid compartment of the vasculature by tumour and non-tumour cells within the tumour microenvironment but also by normal constituents of blood, including erythrocytes, platelets, and leukocytes. Despite the best processing techniques to remove the cellular components of blood, plasma samples are not cell-free. Thus, the levels of miRNAs in plasma are reflective of the miRNAs released from tissues into plasma, any residual cells within plasma, and miRNAs released from lysed blood cells (Pritchard et al., 2012). Erythrocytes, platelets, and leukocytes carry miRNAs and the concentrations of these cells within blood, which can vary depending on disease or physiological state, influence the level of miRNA (Pritchard et al., 2012). MiR-223-p and miR-451a were highly expressed in our plasma samples, and these two miRNAs are enriched in neutrophils and platelets, and erythrocytes, respectively (Fig. 3). Specifically, for miR-451a, there is a difference in its plasma level between the two cases (Fig. 3) and this correlated to the degree of hemolysis in the sample (data not shown). Although potentially relevant to the overall biological process, the high levels (low Ct values) of these miRNAs in plasma likely reflect miRNAs derived from neutrophils, platelets, and erythrocytes rather than the primary tumour.

The plasma miRNAs whose levels indicate production and release by the tumour cells are those that are expressed in both cell lines and tumour tissue. Unfortunately, miRNAs with this pattern of expression still pose difficulty in accurate interpretation of data as many miRNAs are widespread in many different cell types, including neoplastic and circulating cells. For example, the Ct values for all samples for miR-16-5p and miR-19a-3p are within approximately a 2-3-fold difference but both of these miRNAs are also known to have high expression in blood cells (Pritchard et al., 2012). miRNAs such as miR-145-5p and miR-23a still hold promise as they are not found in circulating blood cells and are likely reflective of tumour expression, as they are expressed in both tissues and cell lines to a similar degree. Both of these miRNAs were shown to have various downstream targets that regulate cellular processes involved in cancer such as proliferation and invasion (Gourbault and Llobat, 2020).

### miRNA expression is influenced by cellular proliferation and *in vitro* conditions

The four cell lines investigated had significantly different cell doubling times (Fig. 4) and expression of miR-34a-5p demonstrated a significant correlation with doubling time. It was not expressed in OVC-cOSA-31, which had the fastest doubling time, and had the highest expression in OVC-cMES-103, which had the slowest doubling time and is the only non-cancer cell line. As identified in our target gene analysis, miR-34a-5p targets *MDM4*, and is an important regulator of the tumour suppressor gene *TP53* (Table S4) (Mandke et al., 2012). *MDM4* acts to inhibit transcription and degrade p53 tumour suppressor protein. High expression of miR-34a-5p results in a decrease in *MDM4*, which subsequently leads to maintained expression of p53 tumour suppressor protein (Mandke et al., 2012). A lack of miR-34a-5p, as was found in the quickly dividing cells, could cause a loss of *MDM4* downregulation leading to increased degradation of the p53 tumour suppressor protein and increased proliferation. While HCN3, and FAM76A were also identified as targets of miR-34a-5p, HCN3 did not have a GOnet annotation related directly to cellular proliferation and FAM76A had no annotation in GOnet so the significance of these as miR-34a-5p targets is unknown.

Certain miRNAs exhibited no or limited expression in either all the cell lines or all the tissue samples. The loss of expression of these miRNAs could reflect adaptation to the cell culture environment. MiR-127-3p and miR-379-5p were not expressed in the cell lines but were expressed in tissue. MiR-127-3p induces cellular senescence by targeting BCL6 in human fibroblasts and maintains quiescence in skeletal muscle satellite cells (Castel et al., 2018; Chen et al., 2013). In a study by Castel et al., (2018), miR-127-3p was downregulated in activated myogenic cells compared to quiescent cells, and miR-379-5p had a similar pattern to miR-127-3p in the myogenic cells. Thus, the lack of expression in the cell lines in our study may be related to the adaptation or selection of cells that are highly proliferative in culture. In the current study, we also found that miR-708-5p and miR-224-5p levels were significantly higher in tissues compared to their derived cell lines. In normal tissues, miR-708-5p miRNA inhibits proliferation of satellite cells in syncytial striated muscle and promotes differentiation via its interactions with Notch and Tensin3, as well as other pathways (Baghdadi et al., 2018; Xu et al., 2022). In gastric cancer, miR-224-5p is induced by hypoxia and HIF-1a which inhibits RASSF8 to promote cell growth, migration, and invasion (He et al., 2017). Expression of miR-224-5p was lower in our cell lines compared to tissues, which is likely due to the lack of hypoxic conditions in our cell culture. Finally, miR-182-5p and miR-183-5p had higher expression in the derived cell lines compared to their corresponding tissues, although this was not statistically significant. These miRNAs, along with miR-96-5p are a highly conserved cluster (Dambal et al., 2015). They are enriched in embryonic stem cells, as well as in the eye and ear, and are important in the development of these structures (Dambal et al., 2015). There is a relative lack of these miRNAs in other non-sensory adult tissues, which makes their expression in OS cells somewhat surprising. However, miR-183-5p was also investigated by Castel et al. (2018) and it was absent in quiescent cells but markedly increased in the activated cells. The presence of these miRNAs in embryonic stem cells and activated satellite cells of muscle suggests an important role in proliferation. Although our canine OS tissues did not express miR-183-5p, Jin et al. (2021) demonstrated expression in human OS tissue, thus this difference could reflect species variation. However, in a separate ongoing study, our analysis of a greater number of canine OS tissues has demonstrated marked variation in miR-183-5p expression between dogs (data not shown) and as such the lack of expression in the current study may reflect the low number of samples. Although these sets of patient-matched samples provide a powerful comparison across sample types, a limitation of this study was the availability of such matched samples from only two individuals across all three sample types and three individuals with tissue and derived cell lines, and these comparisons should be expanded to more patients and across tumour types and species.

Comparing patient-matched samples, including tumour tissue, derived cell lines, and blood components, offers unique insights into the biological processes occurring in cancer patients. Obtaining such matched samples can be difficult, especially in rare tumour types such as OS in humans. OS is much more common in dogs and amputation surgery prior to chemotherapy is standard treatment, which makes cell culture and tissue sampling more practical than in humans who receive neoadjuvant chemotherapy and undergo limb-spare procedures. This highlights the utility of canine OS as an important translational model of the human disease (Simpson et al., 2017; Van Der Weyden et al., 2020). miRNAs represent important diagnostic and prognostic biomarkers, as well as potential therapeutic targets in various cancers and are relatively stable and simple to measure in both tissues and bodily fluids. This study highlights the importance of examining matched samples to provide a more complete investigation of the miRNA profiles of cancer patients. Knowledge of the cell types that contribute to the miRNA milieu found in different sample types is crucial for the appropriate interpretation of miRNA expression. miRNAs that are similarly expressed in tissues and cell lines are likely uniquely expressed or dysregulated in neoplastic cells. However, miRNAs that are commonly expressed across many cell types should be considered as well. Integration of miRNA profiles from different sample types can provide important insights into the biology of these master regulators of gene expression and their role in neoplastic diseases and will advance progress toward the development of clinically useful diagnostic tests and therapies.

## MATERIALS AND METHODS

### Samples

#### Tissue samples

All animal experiments were performed according to Canadian Council of Animal Care guidelines. This study was approved by the Animal Care Committee of the University of Guelph, AUP#4409, and consent to collect tissue samples was obtained from the dogs' owners. Tissue samples from two dogs with primary appendicular OS (OVC-cOSA-75 and OVC-cOSA-78) and one dog with pulmonary metastatic OS (OVC-cOSA-31) were obtained from fresh specimens within 1 h of either limb amputation (primary) or euthanasia (metastatic). Representative tissue (approximately 2.0 cm^3^) was collected and rinsed with phosphate-buffered saline (PBS) and immediately and submerged in 1.5-2.0 ml of RNA*later*™ Stabilization Solution (RNAlater; Thermo Fisher Scientific). After 3-5 days at 4°C, the solution was removed, and the tissue was stored in a cryovial at −80°C.

#### Cell lines

The cell lines (OVC-cOSA-31, OVC-cOSA-75, OVC-cOSA-78, and OVC-cMES-103) were derived in-house from the patient samples above and stored in liquid nitrogen as described by Schott et al. (2018). A single vial of each cell line used for miRNA isolation was thawed, expanded, and passaged to reach at least 3 million cells using the conditions described by Schott et al. (2018). Cells were washed with PBS, trypsinized with 1 ml of 0.05% Trypsin with 0.53 mM EDTA (Corning, 25-052-Cl), resuspended in 1 ml of standard media as described by Schott et al. (2018) and transferred to a 15 ml conical tube. The tubes were centrifuged at 1500 rpm at 22°C for 5 min. The media was aspirated, and the cells were resuspended in PBS. The suspension was split into three aliquots of 1 million cells each, then centrifuged at 1500 rpm at 22°C for 5 min. The PBS was aspirated, and the cells were resuspended in 200 µl of RNAlater before being stored at 4°C. Following overnight storage, 1 ml of ice-cold PBS was added, and the aliquots were centrifuged at 1500 rpm at 22°C for 5 min, followed by removal of the supernatant and storage of the cell pellet at −80°C until analysis. OVC-cMES-103 was derived from a limb amputation as part of the standard-of-care treatment for canine OS, however, as described by Schott et al. (2018), is of non-neoplastic mesenchymal origin.

#### Plasma samples

Plasma was available for OVC-cOSA-75 and OVC-cOSA-78 and collected as part of a separate study. Blood samples were collected just prior to limb amputation in K_2_EDTA tubes (BD Vacutainer) and then centrifuged at 1465×***g*** for 10 min at room temperature to separate the plasma. Plasma samples were stored at −80°C until analysis.

### miRNA isolation

miRNAs from the cell lines and tissues were isolated using the QIAGEN miRNeasy Tissue/Cell Advanced Mini Kit. An RNAlater preserved cell pellet of 1 million cells or 14.7-15.0 mg of tissue were used as the starting materials for the isolations. A working buffer was made by adding 100 µl of 2 M dithiothreitol (0.154 g dithiothreitol in 0.5 ml RNase-free water) to 5 ml Buffer RLT, and 260 µl of this working buffer was added to both sample types. For the tissue samples, a stainless-steel bead was added to the tube and the tissue was homogenized at 30 Hz for 4 min using the TissueLyser II (QIAGEN). The bead was removed, and the lysate centrifuged for 3 min at 14,500 rpm (Eppendorf MiniSpin). The supernatant was placed into a new tube without disturbing the pellet. The cell pellet was disrupted in the working buffer by flicking and homogenized by vortexing for 1 min. The remaining steps were the same between the tissue and cell line samples and followed the manufacturer's protocol. The column was transferred to the final 1.5 ml storage tube, 50 µl of RNase-free water was added to the centre of the column, incubated for 1 min at room temperature and centrifuged for 1 min at 14,500 rpm (Eppendorf MiniSpin). 2 µl of the sample was used to quantify the RNA concentration and assess purity (260/280 and 260/230 ratios) using the NanoDrop™ 2000 (Thermo Fisher Scientific). All samples except OVC-cOSA-31 tissue had a concentration of >150 ng/µl, 260/280 ratio>1.9 and 260/230 ratio>1.5. OVC-cOSA-31 tissue had a concentration of 49.8 ng/µl, 260/280 ratio of 1.9, and 260/230 of 1.09. These RNA concentrations were used to calculate input in the subsequent reverse transcription. For the plasma samples, the QIAGEN miRNeasy Serum/Plasma Kit was used with a standard starting input volume of 200 µl per sample. The kit was utilized as per the manufacturer's protocol. As per QIAGEN's recommendations, *cel-*miR-39 was added at the time of isolation to evaluate efficiency of the isolation protocol. The column was transferred to the final 1.5 ml storage tube, and 14 µl of RNase-free water was added to the centre of the column and centrifuged for 1 min at 14,500 rpm (Eppendorf MiniSpin). The sample concentration and purity were not assessed, as the NanoDrop™ 2000 has been deemed unreliable for low RNA concentrations.

### Reverse transcription polymerase chain reaction

For the cell lines and tissue samples, each sample was diluted to 5 ng/µl in nuclease-free water using 2 µl of the miRNA isolate stock. The QIAGEN miRCURY LNA RT kit was used for all samples, following the manufacturer's instructions to obtain 10 µl of cDNA with a standard input of 10 ng of template RNA. A UniSp6 spike-in control was added per QIAGEN's recommendations to evaluate and ensure efficiency. The same kit and final volume were used for the plasma samples, however 0.56 µl of template RNA (equivalent to 8 µl of the original sample) was used. The samples were incubated (Bio-Rad ThermoCycler) at 42°C for 60 min, 95°C for 5 min, and 4°C for at least 5 min before continuing to real-time quantitative polymerase chain reaction (RT-qPCR) (plasma) or stored at −20°C overnight (cell lines and tissues). A no-template control (NTC) was included, replacing the sample volume with water.

### RT-qPCR

The cDNA was diluted at 1:80 for tissue and cell line samples and 1:40 for plasma samples with nuclease-free water and the QIAGEN miRCURY LNA SYBR Green kit was used following the manufacturer's protocol. Custom QIAGEN miRCURY LNA PCR arrays were constructed separately for the tissues/cell lines and plasma samples. 59 miRNAs of interest were analysed for tissues/cell lines, of which 26 were also present on the arrays designed for the previous plasma sample study. These miRNAs were selected based on those described in the literature in canine OS and previous results from analyses using the Canine miRNome array from QIAGEN. A list of the miRNAs analysed is provided in Table S1. The plates were analysed using the Roche LightCycler 480 II. Due to low overall expression in OVC-cMES-103, the cell line was run in duplicate, and the average of each miRNA was used for further analysis. Cycle threshold (Ct) values were obtained by the second derivative maximum method using the LC480 software (release 1.5.1.62 SP3).

### Statistical analysis of Ct values

All Ct values were calibrated by controlling for inter-plate variation with the NTC UniSp3 control for each plate compared to the overall plate average. miRNAs with a Ct value greater than 35 or those with no detection (Ct=0), were set to 35 to provide a conservative estimate of the Ct values and their fold-differences. These fully calibrated values were utilized for further analyses and are subsequently only referred to as Ct values. The average of three miRNAs were used as endogenous controls for normalization of all cell lines and tissue samples (hsa-miR-151a-5p, hsa-let-7b-5p, and hsa-miR-93-5p). The endogenous controls for the cell line and tissue samples were selected using NormFinder (R package) based on cell lines (*n*=4) and tissue (*n*=59, Table S5) samples analysed on the current custom arrays designed for another study and only miRNAs expressed (Ct<35) in all samples were included in the data used for NormFinder analysis (Andersen et al., 2004). Tissues and cell lines were analysed together and separately to select three miRNAs with high stability across all sample types. The endogenous controls (cfa-miR-23b, rno-miR-223-3p, and hsa-miR-27b-3p) for the plasma samples were selected separately as part of a previous study (Ludwig et al., 2024), also utilizing NormFinder (Andersen et al., 2004). The endogenous controls selected for the plasma samples versus cell lines and tissues are markedly different and the amount of input RNA used for the reverse transcription is different. Therefore, only the calibrated Ct values were used for comparisons between sample types as normalized expression values are not comparable. Fold-differences for the tissue and cell line samples were determined using 2^-ΔΔCt^. MiRNAs were considered to have a difference in regulation if the fold-difference was>+/- 2. Paired *t*-tests were used to determine statistical significance (*P<*0.05) when comparing the overall tissue to cell line miRNA profiles. The other comparisons did not contain enough samples per group to perform further statistical analyses.

### Cell line doubling time

An additional vial of each cell line was utilized for the proliferation assay. Cells were thawed and maintained at standard conditions described by Schott et al. (2018). Confluent cells were rinsed with 5 ml of Dulbecco's PBS (Hyclone, SH30028.02), trypsinized using 1 ml of 0.05% Trypsin with 0.53 mM EDTA (Corning, 25-052-CI) and resuspended in 10 ml of standard media to create a single cell suspension. The seeding density that resulted in 100% confluence in approximately 7 days was established for each cell line. Cell density was measured using the TC20 Automated Cell Counter (Bio-Rad) and the suspension was diluted with standard media to result in the appropriate seeding density for each cell line. OS cell lines were seeded in triplicate in 96-well culture microplates (Corning Costar, 3596) and incubated for 24 h under standard conditions to allow attachment. Plates were then transferred into the Agilent BioTek Biospa Automated Incubator maintained under standard conditions, and 3800 µm×3800 µm high contrast brightfield images of the centre of each well were taken every 8 h until 100% confluence was reached using the 4x objective in the Agilent BioTek Cytation C10 Imaging Reader. Media was changed every 4 days after seeding. BioTek Gen5 3.14 software was used to create confluence masks for each cell line. Percent confluence over time was plotted logarithmically to identify a period of exponential cell growth and the linear portion of the curve was used to calculate cell doubling time using R Statistical Software (v.4.2.2; R Core Team, 2022). For each cell line, the experiment was performed twice, and the replicates were averaged for analysis.

### Statistical analysis and gene targets for correlations between miRNA expression and doubling time

A one-way ANOVA followed by post-hoc Tukey's multiple comparisons test was performed to determine if the average doubling time was significantly different between cell lines with a *P*-value cut-off value of <0.05. The average doubling time for each cell line and the normalized Ct value for each miRNA were used to identify correlations between these two parameters. A Pearson correlation coefficient was determined with a two-tailed *P-*value and considered statistically significant if *P*<0.05. Gene targets were evaluated for any miRNAs that statistically correlated with the doubling time of cell lines. Target genes were investigated using TargetScan (https://www.targetscan.org/vert_80/) and miRDB (https://mirdb.org/).

## Supplementary Material

10.1242/biolopen.060483_sup1 Supplementary information

## References

[BIO060483C1] An, T., Fan, T., Zhang, X. Q., Liu, Y.-F., Huang, J., Liang, C., Lv, B.-H., Wang, Y.-Q., Zhao, X.-G., Liu, J.-X. et al. (2019). Comparison of alterations in miRNA expression in matched tissue and blood samples during spinal cord glioma progression. *Sci. Rep.* 9, 9169. 10.1038/s41598-019-42364-x 31235820 PMC6591379

[BIO060483C2] Andersen, C. L., Jensen, J. L. and Ørntoft, T. F. (2004). Normalization of real-time quantitative reverse transcription-PCR data: A model-based variance estimation approach to identify genes suited for normalization, applied to bladder and colon cancer data sets. *Cancer Res.* 64, 5245-5250. 10.1158/0008-5472.CAN-04-0496 15289330

[BIO060483C3] Baghdadi, M. B., Firmino, J., Soni, K., Evano, B., Di Girolamo, D., Mourikis, P., Castel, D. and Tajbakhsh, S. (2018). Notch-induced miR-708 antagonizes satellite cell migration and maintains quiescence. *Cell Stem Cell* 23, 859-868.e5. 10.1016/j.stem.2018.09.017 30416072

[BIO060483C4] Beck, J., Ren, L., Huang, S., Berger, E., Bardales, K., Mannheimer, J., Mazcko, C. and Leblanc, A. (2022). Canine and murine models of osteosarcoma. *Vet. Pathol.* 59, 399-414. 10.1177/03009858221083038 35341404 PMC9290378

[BIO060483C5] Castel, D., Baghdadi, M. B., Mella, S., Gayraud-Morel, B., Marty, V., Cavaillé, J., Antoniewski, C. and Tajbakhsh, S. (2018). Small-RNA sequencing identifies dynamic microRNA deregulation during skeletal muscle lineage progression. *Sci. Rep.* 8, 4208. 10.1038/s41598-018-21991-w 29523801 PMC5844870

[BIO060483C6] Chen, J., Wang, M., Guo, M., Xie, Y. and Cong, Y.-S. (2013). miR-127 regulates cell proliferation and senescence by targeting BCL6. *PLoS One* 8, e80266. 10.1371/journal.pone.0080266 24282530 PMC3840165

[BIO060483C7] Chistiakov, D. A., Orekhov, A. N. and Bobryshev, Y. V. (2016). The role of miR-126 in embryonic angiogenesis, adult vascular homeostasis, and vascular repair and its alterations in atherosclerotic disease. *J. Mol. Cell. Cardiol.* 97, 47-55. 10.1016/j.yjmcc.2016.05.007 27180261

[BIO060483C8] Condrat, C. E., Thompson, D. C., Barbu, M. G., Bugnar, O. L., Boboc, A., Cretoiu, D., Suciu, N., Cretoiu, S. M. and Voinea, S. C. (2020). miRNAs as biomarkers in disease: latest findings regarding their role in diagnosis and prognosis. *Cells* 9, 276. 10.3390/cells9020276 31979244 PMC7072450

[BIO060483C9] Dailey, D. D., Hess, A. M., Bouma, G. J. and Duval, D. L. (2021). MicroRNA expression changes and integrated pathways associated with poor outcome in canine psteosarcoma. *Front. Vet. Sci.* 8, 637622. 10.3389/fvets.2021.637622 33937369 PMC8081964

[BIO060483C10] Dambal, S., Shah, M., Mihelich, B. and Nonn, L. (2015). The microRNA-183 cluster: the family that plays together stays together. *Nucleic Acids Res.* 43, 7173-7188. 10.1093/nar/gkv703 26170234 PMC4551935

[BIO060483C11] Fenger, J. M., Roberts, R. D., Iwenofu, O. H., Bear, M. D., Zhang, X., Couto, J. I., Modiano, J. F., Kisseberth, W. C. and London, C. A. (2016). MiR-9 is overexpressed in spontaneous canine osteosarcoma and promotes a metastatic phenotype including invasion and migration in osteoblasts and osteosarcoma cell lines. *BMC Cancer* 16, 784. 10.1186/s12885-016-2837-5 27724924 PMC5057229

[BIO060483C12] Forterre, A., Komuro, H., Aminova, S. and Harada, M. (2020). A Comprehensive review of cancer microRNA therapeutic delivery strategies. *Cancers* 12, 1852. 10.3390/cancers12071852 32660045 PMC7408939

[BIO060483C13] Geusau, A., Borik–Heil, L., Skalicky, S., Mildner, M., Grillari, J., Hackl, M. and Sunder–Plassmann, R. (2020). Dysregulation of tissue and serum microRNAs in organ transplant recipients with cutaneous squamous cell carcinomas. *Health Sci. Rep.* 3, e205. 10.1002/hsr2.205 33251338 PMC7676459

[BIO060483C14] Gmerek, L., Martyniak, K., Horbacka, K., Krokowicz, P., Scierski, W., Golusinski, P., Golusinski, W., Schneider, A. and Masternak, M. M. (2019). MicroRNA regulation in colorectal cancer tissue and serum. *PLoS One* 14, e0222013. 10.1371/journal.pone.0222013 31469874 PMC6716664

[BIO060483C15] Gourbault, O. and Llobat, L. (2020). MicroRNAs as biomarkers in canine osteosarcoma: a new future? *Vet. Sci.* 7, 146. 10.3390/vetsci7040146 33008041 PMC7711435

[BIO060483C16] Hassan, B. B., Altstadt, L. A., Dirksen, W. P., Elshafae, S. M. and Rosol, T. J. (2020). Canine thyroid cancer: Molecular characterization and cell line growth in nude mice. *Vet. Pathol.* 57, 227-240. 10.1177/0300985819901120 32081094

[BIO060483C17] He, C., Wang, L., Zhang, J. and Xu, H. (2017). Hypoxia-inducible microRNA-224 promotes the cell growth, migration and invasion by directly targeting RASSF8 in gastric cancer. *Mol. Cancer* 16, 35. 10.1186/s12943-017-0603-1 28173803 PMC5297251

[BIO060483C18] Hu, J., Xu, Y., Hao, J., Wang, S., Li, C. and Meng, S. (2012). MiR-122 in hepatic function and liver diseases. *Protein Cell* 3, 364-371. 10.1007/s13238-012-2036-3 22610888 PMC4875471

[BIO060483C19] Jin, L., Luo, Y., Zhao, Y.-C. and Tao, H. (2021). MiR-183-5p promotes tumor progression of osteosarcoma and predicts poor prognosis in patients. *Cancer Manag. Res.* 13, 805-814. 10.2147/CMAR.S285909 33536788 PMC7850385

[BIO060483C20] Keller, A., Gröger, L., Tschernig, T., Solomon, J., Laham, O., Schaum, N., Wagner, V., Kern, F., Schmartz, G. P., Li, Y. et al. (2022). miRNATissueAtlas2: an update to the human miRNA tissue atlas. *Nucleic Acids Res.* 50, D211-D221. 10.1093/nar/gkab808 34570238 PMC8728130

[BIO060483C21] Koenig, E. M., Fisher, C., Bernard, H., Wolenski, F. S., Gerrein, J., Carsillo, M., Gallacher, M., Tse, A., Peters, R., Smith, A. et al. (2016). The beagle dog MicroRNA tissue atlas: identifying translatable biomarkers of organ toxicity. *BMC Genomics* 17, 649. 10.1186/s12864-016-2958-x 27535741 PMC4989286

[BIO060483C22] Kuosmanen, S. M., Kansanen, E., Sihvola, V. and Levonen, A.-L. (2017). MicroRNA profiling reveals distinct profiles for tissue-derived and cultured endothelial cells. *Sci. Rep.* 7, 10943. 10.1038/s41598-017-11487-4 28887500 PMC5591252

[BIO060483C23] Leonardi, L., Scotlandi, K., Pettinari, I., Benassi, M. S., Porcellato, I. and Pazzaglia, L. (2021). MiRNAs in canine and human osteosarcoma: a highlight review on comparative biomolecular aspects. *Cells* 10, 428. 10.3390/cells10020428 33670554 PMC7922516

[BIO060483C24] Lopes-Ramos, C. M., Paulson, J. N., Chen, C.-Y., Kuijjer, M. L., Fagny, M., Platig, J., Sonawane, A. R., Demeo, D. L., Quackenbush, J. and Glass, K. (2017). Regulatory network changes between cell lines and their tissues of origin. *BMC Genomics* 18, 723. 10.1186/s12864-017-4111-x 28899340 PMC5596945

[BIO060483C25] Ludwig, L., Edson, M., Treleaven, H., Viloria-Petit, A. M., Mutsaers, A. J., Moorehead, R., Foster, R. A., Ali, A., Wood, R. D. and Wood, G. A. (2024). Plasma microRNA signatures predict prognosis in canine osteosarcoma patients. *PLoS One*. Public Library of Science (In Press). 10.1371/journal.pone.0311104 PMC11687810 39739708

[BIO060483C26] Mandke, P., Wyatt, N., Fraser, J., Bates, B., Berberich, S. J. and Markey, M. P. (2012). MicroRNA-34a modulates MDM4 expression via a target site in the open reading frame. *PLoS One* 7, e42034. 10.1371/journal.pone.0042034 22870278 PMC3411609

[BIO060483C27] Matsuzaki, J. and Ochiya, T. (2017). Circulating microRNAs and extracellular vesicles as potential cancer biomarkers: a systematic review. *Int. J. Clin. Oncol.* 22, 413-420. 10.1007/s10147-017-1104-3 28243946

[BIO060483C28] Mirabelli, P., Coppola, L. and Salvatore, M. (2019). Cancer cell lines are useful model systems for medical research. *Cancers* 11, 1098. 10.3390/cancers11081098 31374935 PMC6721418

[BIO060483C29] Neudecker, V., Brodsky, K. S., Clambey, E. T., Schmidt, E. P., Packard, T. A., Davenport, B., Standiford, T. J., Weng, T., Fletcher, A. A., Barthel, L. et al. (2017). Neutrophil transfer of *miR-*223 to lung epithelial cells dampens acute lung injury in mice. *Sci. Transl. Med.* 9, eaah5360. 10.1126/scitranslmed.aah5360 28931657 PMC5842431

[BIO060483C30] O'Brien, J., Hayder, H., Zayed, Y. and Peng, C. (2018). Overview of microRNA biogenesis, mechanisms of actions, and circulation. *Front. Endocrinol.* 9, 402. 10.3389/fendo.2018.00402 PMC6085463 30123182

[BIO060483C31] Peng, Y. and Croce, C. M. (2016). The role of microRNAs in human cancer. *Sig. Transduct. Target Ther.* 1, 15004. 10.1038/sigtrans.2015.4 PMC5661652 29263891

[BIO060483C32] Pritchard, C. C., Kroh, E., Wood, B., Arroyo, J. D., Dougherty, K. J., Miyaji, M. M., Tait, J. F. and Tewari, M. (2012). Blood cell origin of circulating microRNAs: A cautionary note for cancer biomarker studies. *Cancer Prev. Res.* 5, 492-497. 10.1158/1940-6207.CAPR-11-0370 PMC4186243 22158052

[BIO060483C33] R Core Team. (2022). R: A language and environment for statistical computing. R Foundation for Statistical Computing, Vienna, Austria. https://www.R-project.org/.

[BIO060483C34] Sahabi, K., Selvarajah, G. T., Abdullah, R., Cheah, Y. K. and Tan, G. C. (2018). Comparative aspects of microRNA expression in canine and human cancers. *J. Vet. Sci.* 19, 162. 10.4142/jvs.2018.19.2.162 28927253 PMC5879064

[BIO060483C35] Sayed, D. and Abdellatif, M. (2011). MicroRNAs in development and disease. *Physiol. Rev.* 91, 827-887. 10.1152/physrev.00006.2010 21742789

[BIO060483C36] Schneider, A., Victoria, B., Lopez, Y. N., Suchorska, W., Barczak, W., Sobecka, A., Golusinski, W., Masternak, M. M. and Golusinski, P. (2018). Tissue and serum microRNA profile of oral squamous cell carcinoma patients. *Sci. Rep.* 8, 675. 10.1038/s41598-017-18945-z 29330429 PMC5766573

[BIO060483C37] Schott, C. R., Ludwig, L., Mutsaers, A. J., Foster, R. A. and Wood, G. A. (2018). The autophagy inhibitor spautin-1, either alone or combined with doxorubicin, decreases cell survival and colony formation in canine appendicular osteosarcoma cells. *PLoS One* 13, e0206427. 10.1371/journal.pone.0206427 30372478 PMC6205606

[BIO060483C38] Simpson, S., Dunning, M. D., De Brot, S., Grau-Roma, L., Mongan, N. P. and Rutland, C. S. (2017). Comparative review of human and canine osteosarcoma: morphology, epidemiology, prognosis, treatment and genetics. *Acta. Vet. Scand.* 59, 71. 10.1186/s13028-017-0341-9 29065898 PMC5655853

[BIO060483C39] Singh, V. M., Salunga, R. C., Huang, V. J., Tran, Y., Erlander, M., Plumlee, P. and Peterson, M. R. (2013). Analysis of the effect of various decalcification agents on the quantity and quality of nucleic acid (DNA and RNA) recovered from bone biopsies. *Ann. Diagn. Pathol.* 17, 322-326. 10.1016/j.anndiagpath.2013.02.001 23660273

[BIO060483C40] Smolarz, B., Durczyński, A., Romanowicz, H., Szyłło, K. and Hogendorf, P. (2022). miRNAs in cancer (review of literature). *Int. J. Mol. Sci.* 23, 2805. 10.3390/ijms23052805 35269947 PMC8910953

[BIO060483C41] Thakur, S., Grover, R. K., Gupta, S., Yadav, A. K. and Das, B. C. (2016). Identification of specific miRNA signature in paired sera and tissue samples of Indian women with triple negative breast cancer. *PLoS One* 11, e0158946. 10.1371/journal.pone.0158946 27404381 PMC4942139

[BIO060483C42] Van Der Weyden, L., Starkey, M., Abu-Helil, B., Mutsaers, A. J. and Wood, G. A. (2020). Companion canines: an under-utilised model to aid in translating anti-metastatics to the clinic. *Clin. Exp. Metastasis* 37, 7-12. 10.1007/s10585-019-10002-5 31691156 PMC7007897

[BIO060483C43] Van Roosbroeck, K. and Calin, G. A. (2017). Cancer hallmarks and microRNAs: The therapeutic connection. *Adv. Cancer Res.* 135, 119-149. 10.1016/bs.acr.2017.06.002 28882220

[BIO060483C44] Witwer, K. W. and Halushka, M. K. (2016). Toward the promise of microRNAs – enhancing reproducibility and rigor in microRNA research. *RNA Biol.* 13, 1103-1116. 10.1080/15476286.2016.1236172 27645402 PMC5100345

[BIO060483C45] Xu, X., Lu, H., Xu, D., Yu, Z., Ai, N., Wang, K., Li, X., He, J., Jiang, J., Ma, H. et al. (2022). miR-708-5p regulates myoblast proliferation and differentiation. *Vet. Sci.* 9, 641. 10.3390/vetsci9110641 36423090 PMC9694511

