# Supplementary material for: Patient-matched tumours, plasma, and cell lines reveal tumour microenvironment- and cell culture-specific microRNAs
Source: Biol Open. 2024 Dec 23;13(12):bio060483. doi: 10.1242/bio.060483 (PMC11695573; doi:10.1242/bio.060483)
Supplement: Supplementary information [file biolopen-13-060483-s1.pdf]

Table S1. List of miRNAs on the tissue array, serum array, and those that underwent final analysis after quality control.

miRNA	Tissue/cell array	Serum array	Included in analysis
hsa-miR-379-5p	Y		Y
hsa-130a-3p	Y		Y
hsa-miR-326	Y		
hsa-miR-185-5p	Y	Y	Y
hsa-miR-378a-3p	Y	Y	Y
cfa-miR-221	Y	Y	Y
hsa-miR-335-5p	Y		Y
hsa-miR-22-3p	Y	Y	Y
hsa-miR-16-5p	Y	Y	Y
hsa-miR-145-5p	Y	Y	Y
bta-miR-99a-5p	Y		Y
hsa-miR-885-5p	Y	Y	Y
hsa-miR-452-5p	Y		Y
hsa-miR-542-3p	Y		Y
hsa-miR-9-5p	Y		Y
hsa-miR-95-3p	Y		Y
cfa-miR-1844	Y		
rno-miR-223-3p	Y	Y	Y
hsa-miR-34a-5p	Y		Y
rno-miR-224-5p	Y		Y
hsa-miR-182-5p	Y		Y
cfa-miR-142	Y	Y	Y
hsa-miR-200c-3p	Y		Y
hsa-miR-126-5p	Y	Y	Y
hsa-miR-127-3p	Y		Y
hsa-miR-151a-5p	Y	Y	Y
hsa-miR-708-5p	Y		Y
mmu-miR-592-5p	Y		Y
hsa-miR-218-5p	Y		Y
cfa-miR-133c	Y	Y	Y
hsa-miR-206	Y		
hsa-miR-128-3p	Y	Y	Y
hsa-miR-143-3p	Y	Y	Y
hsa-miR-150-5p	Y		Y
hsa-miR-125b-5p	Y	Y	Y

Biology Open • Supplementary information

hsa-let-7b-5p	Y		Y
cfa-miR-23a	Y	Y	Y
hsa-miR-204-5p	Y		Y
mmu-miR-211-5p	Y		
hsa-miR-196a-5p	Y		Y
cfa-miR-30a	Y	Y	Y
hsa-miR-19a-3p	Y	Y	Y
hsa-miR-29b-3p	Y		Y
hsa-miR-451a	Y	Y	Y
hsa-miR-146b-5p	Y		Y
hsa-miR-7-5p	Y	Y	Y
cfa-miR-1	Y	Y	Y
hsa-miR-362-5p	Y		Y
cfa-miR-1271	Y	Y	Y
hsa-miR-93-5p	Y	Y	Y
hsa-miR-214-3p	Y	Y	Y
hsa-miR-183-5p	Y		Y
cfa-miR-196b	Y		Y
cfa-miR-181b	Y		Y
hsa-miR-27b-3p	Y	Y	Y
hsa-miR-20a-5p	Y	Y	Y
gga-miR-30c-5p	Y		Y
hsa-miR-96-5p	Y		Y
UniSp6	Y	Y	
UniSp3	Y	Y	
UniSp3 (NTC)	Y	Y	
cel-miR-39		Y	

Table S2. miRNAs with no fold-difference (< +/- 2) on average between tissues and cell lines.

miRNA	Average (n = 3)	OVC-cOSA-75	OVC-cOSA-78	OVC-cOSA-31	*P*-value of average
hsa-miR-335-5p	1.97	-1.46	1.22	**9.10**	0.481348
hsa-miR-542-3p	1.89	**2.61**	-1.07	**2.80**	0.216543
cfa-miR-181b	1.80	-1.31	**2.08**	**3.66**	0.329892
gga-miR-30c-5p*	1.73	1.88	1.49	1.84	0.018556
hsa-miR-378a-3p	1.71	**2.13**	1.25	1.87	0.079784
hsa-miR-27b-3p	1.69	**3.95**	**-2.03**	**2.47**	0.497059
hsa-miR-214-3p	1.44	1.55	**2.39**	-1.24	0.367762

hsa-let-7b-5p*	1.44	1.82	1.26	1.30	0.092686
hsa-miR-200c-3p	1.34	-1.42	-1.93	**6.66**	0.747950
hsa-miR-95-3p*	1.33	1.42	1.79	-1.07	0.270989
hsa-miR-885-5p*	1.29	1.01	1.26	1.68	0.270989
bta-miR-99a-5p	1.25	**3.10**	1.13	-1.79	0.699031
hsa-miR-34a-5p	1.25	**2.15**	**-4.30**	**3.88**	0.823593
hsa-miR-130a-3p	1.02	**2.65**	-1.83	-1.34	0.969588
hsa-miR-19a-3p	1.01	**3.82**	-1.33	**-2.80**	0.967515
cfa-miR-196b	1.01	**3.82**	-1.33	**-2.80**	0.995333
hsa-miR-151a-5p*	-1.04	1.13	-1.29	1.00	0.716419
hsa-miR-22-3p	-1.10	**3.35**	**-3.42**	-1.29	0.905936
cfa-miR-23a	-1.10	**-2.73**	-1.30	**2.68**	0.882666
hsa-miR-185-5p*	-1.20	1.02	-1.72	-1.02	0.415949
cfa-miR-101	-1.29	-1.03	**-2.68**	1.28	0.560936
hsa-miR-20a-5p	-1.32	1.13	-1.30	**-2.01**	0.355228
cfa-miR-1	-1.38	-1.40	**-5.45**	**2.92**	0.724603
hsa-miR-93-5p	-1.38	**-2.05**	1.02	-1.30	0.270120
hsa-miR-125b-5p	-1.50	1.47	**-3.28**	-1.51	0.462963
hsa-miR-29b-3p	-1.72	**3.15**	**-4.33**	**-3.72**	0.584151
cfa-miR-133c	-1.83	**-3.00**	**-7.65**	**3.75**	0.605058
hsa-miR-146b-5p	-1.99	1.61	-1.30	**-9.78**	0.492965

For individual comparisons, those with a fold-change of > +/-2 are bolded.
*Indicates those that have no fold-change across all three patients.

Table S3. Calibrated cycle threshold (Ct) values for the derived cell line, tissue, and plasma sample of OVC-cOSA-75 and OVC-cOSA-78.

miRNA	OVC-cOSA-75			OVC-cOSA-78		
	**Cell line**	**Tissue**	**Plasma**	**Cell line**	**Tissue**	**Plasma**
hsa-miR-185-5p	29.96	29.42	35.00	30.12	29.96	34.08
hsa-378a-3p	29.52	27.91	33.45	29.41	28.14	32.73
cfa-miR-221	27.26	26.98	35.00	27.08	29.13	34.75
hsa-miR-22-3p	29.66	27.40	34.00	28.29	29.12	32.78
hsa-miR-16-5p	25.70	24.64	25.00	26.46	25.24	24.59
hsa-miR-145-5p	27.14	25.28	28.31	26.25	26.01	28.49
hsa-miR-885-5p	35.00	34.47	35.00	35.00	33.72	34.85
rno-miR-223-3p	35.00	31.35	26.40	34.98	31.87	25.54
cfa-miR-142	35.00	31.01	29.69	35.00	32.65	28.52
hsa-miR-126-5p	35.00	29.47	31.74	35.00	31.00	29.89
hsa-miR-151a-5p	29.44	28.75	32.46	29.37	28.79	32.41

Biology Open • Supplementary information

cfa-miR-133c	33.06	34.13	32.60	33.01	35.00	33.20
hsa-miR-128-3p	34.20	32.83	35.00	34.01	31.44	31.37
hsa-miR-143-3p	29.21	27.59	34.12	29.33	27.37	32.92
hsa-miR-125b-5p	23.67	22.60	32.10	23.78	24.55	31.34
cfa-miR-23a	24.44	25.37	29.01	25.32	24.76	28.11
cfa-miR-30a	29.31	27.75	35.00	29.23	31.97	32.96
hsa-miR-19a-3p	28.16	27.86	29.69	28.90	27.36	29.23
hsa-miR-451a	35.00	26.68	24.88	35.00	28.95	22.97
hsa-miR-7-5p	30.23	33.52	33.30	30.42	30.75	31.61
cfa-miR-1	34.94	34.91	33.88	33.50	35.00	35.00
cfa-miR-1271	31.66	33.30	35.00	31.40	31.09	35.00
hsa-miR-93-5p	27.40	27.92	31.31	28.36	27.39	30.22
hsa-miR-214-3p	27.12	25.97	35.00	27.42	25.22	31.37
hsa-miR-27b-3p	27.18	24.68	30.42	25.47	25.55	29.42
hsa-miR-20a-5p	28.54	27.85	30.70	28.26	27.69	29.81

Table S4. Top five genes identified for miR-34a-5p using TargetScan and miRDB and their associated biological processes identified with GOnet.

Gene target<br>TargetScan	Biological process – GOnet annotations
MDM4* (Mdm4 p53 binding protein homolog)	mitotic cell cycle, signal transduction, cellular protein modification, protein-containing complex assembly, response to stress, cell cycle, cellular component assembly, cell population proliferation, anatomical development
HCN3* (hyperpolarization activated cyclic nucleotide-gated potassium channel 3)	transmembrane transport, transport
FAM76A* (family with sequence similarity 76, member A)	N/D
SCN2B (sodium channel, voltage-gated, type II, beta subunit)	cell-cell signaling, circulatory system process, anatomical structure development, transmembrane transport, transport, homeostatic process
SYT1 (synaptotagmin I)	protein-containing complex assembly, vesicle-mediated transport, cell-cell signalling, cell differentiation, cellular component assembly, membrane organization, anatomical structure development, transport
miRDB	
HCN3*	transmembrane transport, transport
FAM76A*	N/D

MDM4*	mitotic cell cycle, signal transduction, cellular protein modification, protein-containing complex assembly, response to stress, cell cycle, cellular component assembly, cell population proliferation, anatomical development
RAP1GDS1 (Rap1 GTPase-GDP dissociation stimulator 1)	protein-containing complex assembly, cytoskeleton organization, cellular component assembly, circulatory system process, homeostatic process
FAM167A (family with sequence similarity 167, member A)	N/D

*MDM4, HCN3, and FAM76A were identified in both databases.

Table S5. Calibrated Ct values of cell lines and tissues used to select cell line endogenous controls in NormFinder

miRNA	OVC-cMES-103 - replicate 1	OVC-cMES-103 - replicate 2	OVC-cOSA-31	OVC-cOSA-75	OVC-cOSA-78	OSA_1
hsa-miR-130a-3p	30.66	30.38	30.04	31.26	29.88	29.68
hsa-miR-185-5p	29.71	29.83	29.70	29.96	30.12	29.73
hsa-378a-3p	28.13	29.24	30.12	29.52	29.41	29.86
cfa-miR-221	24.96	25.53	27.73	27.26	27.08	27.35
hsa-miR-22-3p	28.55	28.75	28.96	29.66	28.29	26.37
hsa-miR-16-5p	26.00	26.24	26.98	25.70	26.46	23.04
hsa-miR-145-5p	25.10	25.88	33.49	27.14	26.25	25.72
bta-miR-99a-5p	26.82	27.18	26.42	28.44	28.46	26.01
hsa-miR-151a-5p	30.07	29.32	30.37	29.44	29.37	28.98
cfa-miR-101	30.55	31.22	31.29	30.04	29.83	28.61
hsa-miR-125b-5p	23.98	24.06	24.31	23.67	23.78	22.74
hsa-let-7b-5p	25.61	25.71	27.25	25.89	26.80	24.16
cfa-miR-23a	25.74	25.21	27.61	24.44	25.32	25.24
cfa-miR-30a	31.53	29.95	29.80	29.31	29.23	29.54
hsa-miR-19a-3p	28.84	28.86	27.22	28.16	28.90	25.88
hsa-miR-29b-3p	30.37	31.16	31.94	31.99	31.03	28.13
hsa-miR-7-5p	31.86	31.75	31.86	30.23	30.42	31.55
hsa-miR-362-5p	33.04	32.95	31.71	32.83	31.90	31.63
cfa-miR-1271	32.56	32.23	30.91	31.66	31.40	32.45
hsa-miR-93-5p	28.86	28.32	28.15	27.40	28.36	26.81
hsa-miR-214-3p	27.15	27.00	25.89	27.12	27.42	26.86

cfa-miR-181b	33.77	33.05	31.00	29.27	29.58	27.47
hsa-miR-27b-3p	25.91	26.21	27.47	27.18	25.47	24.93
hsa-miR-20a-5p	28.40	28.28	26.74	28.54	28.26	26.13
gga-miR-30c-5p	29.64	28.89	29.34	29.16	29.05	27.75

miRNA	OSA_2	OSA_3	OSA_4	OSA_5	OSA_6	OSA_7
hsa-miR-130a-3p	30.52	29.96	30.83	29.65	30.48	29.70
hsa-miR-185-5p	30.33	29.93	31.04	30.51	29.64	29.28
hsa-378a-3p	27.97	26.99	28.24	27.94	27.92	26.99
cfa-miR-221	28.44	27.66	29.83	26.81	28.46	29.93
hsa-miR-22-3p	27.28	26.67	27.88	26.07	26.79	27.29
hsa-miR-16-5p	23.83	23.83	24.02	24.32	24.46	24.10
hsa-miR-145-5p	25.54	26.00	25.42	25.23	24.61	24.97
bta-miR-99a-5p	26.42	25.75	25.24	25.99	25.39	25.11
hsa-miR-151a-5p	29.08	28.26	28.13	27.78	28.73	28.39
cfa-miR-101	28.26	28.71	30.26	29.14	29.13	28.09
hsa-miR-125b-5p	23.50	23.31	25.33	25.49	23.90	24.68
hsa-let-7b-5p	24.85	24.14	25.46	25.16	24.65	26.12
cfa-miR-23a	25.82	24.98	26.08	24.48	24.90	25.40
cfa-miR-30a	29.82	29.16	29.37	29.29	28.93	30.03
hsa-miR-19a-3p	27.00	25.37	26.79	27.39	27.38	26.74
hsa-miR-29b-3p	29.68	29.66	31.97	29.67	29.70	29.68
hsa-miR-7-5p	32.26	30.90	31.84	31.28	32.52	31.31
hsa-miR-362-5p	29.79	29.60	28.68	30.97	31.32	29.52
cfa-miR-1271	32.91	31.25	32.63	32.43	32.70	31.46

Biology Open • Supplementary information

hsa-miR-93-5p	28.37	26.66	26.73	25.82	28.23	27.93
hsa-miR-214-3p	25.71	25.44	25.70	25.73	25.60	25.73
cfa-miR-181b	28.97	26.95	29.03	28.47	29.82	29.52
hsa-miR-27b-3p	25.48	24.96	26.39	24.06	25.29	23.98
hsa-miR-20a-5p	27.80	25.78	27.10	26.97	27.10	26.79
gga-miR-30c-5p	27.75	26.84	27.51	27.72	27.44	27.70

miRNA	OSA_8	OSA_9	OSA_10	OSA_11	OSA_12	OSA_13
hsa-miR-130a-3p	28.75	29.05	29.37	29.35	29.35	30.56
hsa-miR-185-5p	29.41	30.71	29.17	29.57	29.47	30.06
hsa-378a-3p	25.77	26.88	28.05	27.47	27.68	28.14
cfa-miR-221	25.92	26.72	26.33	28.55	27.64	29.77
hsa-miR-22-3p	25.94	26.81	27.06	27.43	26.33	27.93
hsa-miR-16-5p	23.48	23.45	24.06	23.63	23.84	24.35
hsa-miR-145-5p	23.72	24.55	25.09	24.12	24.35	24.32
bta-miR-99a-5p	25.38	25.72	25.37	26.52	26.12	26.58
hsa-miR-151a-5p	27.61	28.15	28.84	28.17	28.60	28.68
cfa-miR-101	27.82	30.48	28.59	29.95	29.99	30.51
hsa-miR-125b-5p	23.68	25.60	24.11	25.06	22.68	24.13
hsa-let-7b-5p	24.16	25.36	25.00	25.88	24.02	24.62
cfa-miR-23a	25.59	26.49	25.14	25.69	25.15	26.06
cfa-miR-30a	28.04	30.02	29.99	28.08	29.62	30.08
hsa-miR-19a-3p	26.41	26.13	27.33	25.93	26.86	27.70
hsa-miR-29b-3p	31.29	31.21	31.44	29.94	30.35	31.96
hsa-miR-7-5p	30.93	29.48	31.29	29.10	31.98	33.62

Biology Open • Supplementary information

hsa-miR-362-5p	31.02	30.81	31.53	30.68	30.07	30.64
cfa-miR-1271	30.47	31.17	31.50	31.76	31.90	33.08
hsa-miR-93-5p	27.02	27.54	28.00	27.59	27.45	28.93
hsa-miR-214-3p	25.38	25.91	25.79	26.47	25.92	26.11
cfa-miR-181b	27.69	28.99	28.89	29.35	28.32	31.57
hsa-miR-27b-3p	24.21	25.30	25.51	24.74	24.85	25.69
hsa-miR-20a-5p	26.82	27.04	27.78	26.13	26.68	27.50
gga-miR-30c-5p	26.54	26.69	27.68	27.59	27.86	27.48

miRNA	OSA_14	OSA_15	OSA_16	OSA_17	OSA_18	OSA_19
hsa-miR-130a-3p	28.93	30.91	29.32	29.34	31.06	28.78
hsa-miR-185-5p	29.31	31.35	29.55	29.42	30.65	28.78
hsa-378a-3p	26.14	27.43	27.75	27.91	28.01	28.64
cfa-miR-221	27.83	30.50	28.68	26.98	29.90	24.93
hsa-miR-22-3p	27.44	27.05	29.96	27.40	29.18	26.38
hsa-miR-16-5p	23.32	25.10	25.00	24.64	25.92	24.31
hsa-miR-145-5p	26.10	26.56	26.52	25.28	25.05	25.14
bta-miR-99a-5p	26.00	27.81	26.99	26.29	27.97	26.39
hsa-miR-151a-5p	28.74	30.35	28.70	28.75	30.41	28.33
cfa-miR-101	28.99	31.58	30.03	29.56	29.94	29.03
hsa-miR-125b-5p	25.34	25.88	25.80	22.60	25.02	22.10
hsa-let-7b-5p	24.97	25.66	27.52	24.51	26.41	24.63
cfa-miR-23a	25.90	26.89	26.39	25.37	26.09	23.92
cfa-miR-30a	29.96	29.76	31.26	27.75	29.11	29.07
hsa-miR-19a-3p	26.43	27.47	26.79	27.86	28.73	27.90

hsa-miR-29b-3p	30.85	31.81	32.01	29.82	32.85	28.33
hsa-miR-7-5p	30.33	32.90	29.97	33.52	34.11	32.96
hsa-miR-362-5p	28.96	31.18	30.01	30.00	31.83	31.24
cfa-miR-1271	31.38	32.31	32.26	33.30	33.78	31.17
hsa-miR-93-5p	25.63	28.37	26.85	27.92	29.33	27.53
hsa-miR-214-3p	25.74	26.35	25.66	25.97	27.36	26.09
cfa-miR-181b	28.93	30.80	28.37	29.14	31.67	28.16
hsa-miR-27b-3p	25.47	26.64	25.81	24.68	26.01	23.81
hsa-miR-20a-5p	25.50	27.68	26.67	27.85	29.05	27.81
gga-miR-30c-5p	27.60	28.82	28.17	27.73	28.93	27.37

miRNA	OSA_20	OSA_21	OSA_22	OSA_23	OSA_24	OSA_25
hsa-miR-130a-3p	29.81	30.18	30.68	29.61	30.14	30.90
hsa-miR-185-5p	29.96	28.54	30.33	28.35	30.27	30.37
hsa-378a-3p	28.14	26.34	28.00	26.84	28.68	28.48
cfa-miR-221	29.13	26.30	28.05	25.22	28.30	28.90
hsa-miR-22-3p	29.12	27.74	28.69	27.98	27.33	28.50
hsa-miR-16-5p	25.24	23.75	23.62	23.92	23.32	24.94
hsa-miR-145-5p	26.01	26.30	27.28	24.71	24.02	26.76
bta-miR-99a-5p	27.34	26.75	27.84	25.09	24.10	25.81
hsa-miR-151a-5p	28.79	28.91	30.18	28.09	27.92	29.16
cfa-miR-101	30.31	30.07	30.93	28.22	28.01	30.49
hsa-miR-125b-5p	24.55	25.83	25.14	24.15	22.66	24.49
hsa-let-7b-5p	25.52	26.09	26.52	24.55	24.55	25.60
cfa-miR-23a	24.76	25.16	25.25	24.97	24.16	24.47

cfa-miR-30a	31.97	29.67	29.13	29.35	29.85	31.66
hsa-miR-19a-3p	27.36	27.54	27.63	25.94	27.38	27.53
hsa-miR-29b-3p	32.20	30.57	30.97	29.57	31.75	32.91
hsa-miR-7-5p	30.75	30.66	31.68	31.12	32.67	31.41
hsa-miR-362-5p	29.99	30.71	31.97	29.20	30.37	29.17
cfa-miR-1271	31.09	32.38	32.10	31.65	31.87	32.30
hsa-miR-93-5p	27.39	27.57	26.88	26.16	28.12	27.29
hsa-miR-214-3p	25.22	26.79	27.63	25.86	25.72	25.96
cfa-miR-181b	27.58	28.30	28.76	28.99	27.80	28.62
hsa-miR-27b-3p	25.55	25.82	26.01	24.39	24.12	24.83
hsa-miR-20a-5p	27.69	27.12	27.61	26.21	27.03	27.39
gga-miR-30c-5p	27.53	27.67	27.82	26.73	26.13	27.98

miRNA	OSA_26	OSA_27	OSA_28	OSA_29	OSA_30	OSA_31
hsa-miR-130a-3p	30.04	28.66	30.63	29.92	29.31	29.06
hsa-miR-185-5p	27.55	28.57	30.87	30.60	28.95	29.14
hsa-378a-3p	26.77	27.67	29.06	28.27	27.88	26.96
cfa-miR-221	25.68	26.98	28.68	27.88	26.89	27.84
hsa-miR-22-3p	27.69	26.75	27.98	28.74	26.52	25.70
hsa-miR-16-5p	23.90	23.67	24.40	24.41	23.78	25.14
hsa-miR-145-5p	26.38	23.91	26.60	26.30	23.36	24.16
bta-miR-99a-5p	25.45	25.92	27.29	27.26	24.12	25.54
hsa-miR-151a-5p	27.68	28.13	29.63	27.84	26.76	26.73
cfa-miR-101	29.46	28.26	30.37	30.62	27.07	28.56
hsa-miR-125b-5p	24.79	23.27	25.48	25.58	20.00	23.60

Biology Open • Supplementary information

hsa-let-7b-5p	24.93	24.31	25.87	25.59	24.02	24.51
cfa-miR-23a	25.36	24.62	25.93	24.39	23.69	24.43
cfa-miR-30a	28.32	27.68	29.69	29.94	27.06	29.53
hsa-miR-19a-3p	26.13	26.37	27.05	27.34	27.27	26.06
hsa-miR-29b-3p	31.40	28.36	31.41	32.43	30.53	28.75
hsa-miR-7-5p	29.89	30.71	31.16	30.54	33.60	29.47
hsa-miR-362-5p	27.50	27.69	31.43	30.43	31.71	29.32
cfa-miR-1271	30.91	30.44	34.11	32.51	30.96	31.04
hsa-miR-93-5p	26.25	25.43	28.31	26.69	27.59	26.55
hsa-miR-214-3p	25.25	24.45	26.64	25.71	25.85	25.41
cfa-miR-181b	27.56	28.29	28.93	27.85	26.82	27.63
hsa-miR-27b-3p	24.86	23.08	25.75	25.84	23.75	24.48
hsa-miR-20a-5p	26.44	26.05	27.04	26.27	27.09	26.25
gga-miR-30c-5p	26.63	26.48	28.16	27.74	26.46	27.15

miRNA	OSA_32	OSA_33	OSA_34	OSA_35	OSA_36	metOSA_37
hsa-miR-130a-3p	29.17	30.86	30.65	29.30	31.90	29.70
hsa-miR-185-5p	29.48	28.50	29.10	29.90	31.64	28.85
hsa-378a-3p	28.37	28.34	27.17	28.30	28.39	26.82
cfa-miR-221	27.38	27.97	27.97	28.25	30.86	27.52
hsa-miR-22-3p	28.13	27.52	28.10	27.64	28.78	29.39
hsa-miR-16-5p	24.06	23.01	22.53	24.63	27.03	24.05
hsa-miR-145-5p	25.03	26.83	24.81	25.52	25.00	26.41
bta-miR-99a-5p	24.91	26.30	26.56	25.26	27.41	26.14
hsa-miR-151a-5p	27.85	29.33	28.29	29.01	29.78	28.45

cfa-miR-101	27.89	29.68	29.46	29.47	30.43	30.01
hsa-miR-125b-5p	21.01	24.43	25.25	23.97	24.43	22.90
hsa-let-7b-5p	24.77	26.08	25.70	23.67	26.36	25.88
cfa-miR-23a	24.68	25.15	25.60	25.11	26.43	25.04
cfa-miR-30a	29.26	29.62	29.54	28.72	29.61	28.91
hsa-miR-19a-3p	27.27	26.79	25.62	27.69	29.69	24.84
hsa-miR-29b-3p	30.54	30.64	30.68	30.59	33.41	30.49
hsa-miR-7-5p	32.30	31.88	30.30	32.76	34.00	32.15
hsa-miR-362-5p	31.76	29.27	30.68	30.80	31.69	29.69
cfa-miR-1271	32.13	33.06	32.40	32.60	32.70	31.60
hsa-miR-93-5p	27.65	27.34	26.56	28.02	29.25	25.46
hsa-miR-214-3p	27.04	24.87	26.61	26.07	26.48	26.72
cfa-miR-181b	27.58	26.94	29.70	29.09	32.09	27.84
hsa-miR-27b-3p	24.81	24.76	25.88	24.70	26.55	25.50
hsa-miR-20a-5p	27.39	26.65	26.42	27.82	29.01	24.94
gga-miR-30c-5p	27.11	27.64	27.01	27.34	29.06	27.90

miRNA	metOSA_38	OSA_39	metOSA_40	OSA_41	metOSA_42	metOSA_43
hsa-miR-130a-3p	29.37	29.38	28.51	31.93	30.37	30.63
hsa-miR-185-5p	29.37	29.37	28.87	30.60	29.63	30.82
hsa-378a-3p	28.04	26.51	28.68	29.61	29.12	27.41
cfa-miR-221	26.30	25.53	26.35	27.78	29.00	28.37
hsa-miR-22-3p	27.40	26.41	26.99	28.33	29.23	28.77
hsa-miR-16-5p	23.44	23.10	23.42	25.60	24.34	24.44
hsa-miR-145-5p	23.97	24.08	23.87	25.01	26.69	26.90

Biology Open • Supplementary information

bta-miR-99a-5p	25.56	26.15	25.80	27.44	27.16	27.94
hsa-miR-151a-5p	27.77	27.59	27.28	29.39	30.27	28.23
cfa-miR-101	29.52	28.41	27.92	29.23	30.84	29.07
hsa-miR-125b-5p	23.46	23.30	24.41	25.02	24.81	25.67
hsa-let-7b-5p	24.85	24.58	24.26	24.83	26.78	26.16
cfa-miR-23a	25.24	23.67	24.59	25.65	26.09	24.94
cfa-miR-30a	29.46	28.23	27.75	30.89	32.46	29.69
hsa-miR-19a-3p	26.85	26.55	26.07	28.87	27.45	26.29
hsa-miR-29b-3p	30.00	30.24	28.58	31.87	33.74	29.26
hsa-miR-7-5p	31.99	30.34	29.69	33.68	31.27	29.97
hsa-miR-362-5p	30.43	29.93	30.05	30.76	30.67	31.62
cfa-miR-1271	32.80	31.71	32.71	32.38	31.29	32.30
hsa-miR-93-5p	26.76	27.30	27.26	29.00	28.43	25.20
hsa-miR-214-3p	25.99	26.41	27.75	26.88	26.10	26.58
cfa-miR-181b	27.22	26.49	27.06	28.61	29.03	30.08
hsa-miR-27b-3p	26.01	24.56	24.51	25.73	26.07	25.29
hsa-miR-20a-5p	27.74	26.39	26.14	28.75	27.65	26.63
gga-miR-30c-5p	27.64	26.12	26.57	28.90	28.36	27.70

miRNA	metOSA_44	metOSA_45	metOSA_46	metOSA_47	metOSA_48	metOSA_49
hsa-miR-130a-3p	30.04	28.68	29.11	29.09	27.74	29.93
hsa-miR-185-5p	29.17	28.70	29.91	29.83	29.40	30.03
hsa-378a-3p	27.50	26.80	28.41	28.67	26.83	27.88
cfa-miR-221	25.87	26.71	28.65	28.36	27.75	28.64
hsa-miR-22-3p	28.78	27.73	28.16	27.16	27.30	27.71

Biology Open • Supplementary information

hsa-miR-16-5p	23.92	23.84	24.19	23.65	22.72	24.58
hsa-miR-145-5p	24.36	23.64	24.71	24.08	23.61	24.90
bta-miR-99a-5p	26.80	28.79	26.33	26.36	25.76	24.84
hsa-miR-151a-5p	27.38	27.16	27.97	28.29	27.37	28.37
cfa-miR-101	27.27	29.35	28.14	28.20	27.74	27.59
hsa-miR-125b-5p	22.66	26.00	25.41	24.16	24.41	23.68
hsa-let-7b-5p	24.79	25.92	24.91	25.15	23.65	23.72
cfa-miR-23a	23.91	25.90	25.15	25.78	24.99	25.03
cfa-miR-30a	28.66	29.86	27.94	30.06	26.86	28.12
hsa-miR-19a-3p	25.81	25.73	25.93	26.76	26.11	26.36
hsa-miR-29b-3p	29.31	30.67	29.27	28.64	29.15	28.93
hsa-miR-7-5p	29.63	29.93	32.09	29.48	31.67	29.72
hsa-miR-362-5p	29.93	31.51	30.96	30.74	29.81	27.80
cfa-miR-1271	31.06	31.28	31.88	31.63	32.29	31.43
hsa-miR-93-5p	25.85	26.99	26.84	26.67	26.20	26.11
hsa-miR-214-3p	25.72	28.91	25.75	27.02	28.05	25.64
cfa-miR-181b	26.48	29.19	28.12	28.57	27.88	27.27
hsa-miR-27b-3p	23.64	26.36	24.87	25.19	24.79	24.35
hsa-miR-20a-5p	25.71	25.69	26.06	26.22	26.43	25.70
gga-miR-30c-5p	25.85	27.38	26.57	27.87	26.14	26.71

miRNA	metOSA_50	OSA_51	OSA_52	Lung_53	Lung_54	Lung_55
hsa-miR-130a-3p	29.94	30.31	30.67	28.58	28.93	27.84
hsa-miR-185-5p	29.92	31.10	29.37	29.79	29.37	29.65
hsa-378a-3p	27.60	29.21	28.14	30.23	28.63	29.70

Biology Open • Supplementary information

cfa-miR-221	28.61	30.99	28.75	29.27	28.49	30.44
hsa-miR-22-3p	28.65	28.12	28.86	28.64	28.30	29.39
hsa-miR-16-5p	24.16	26.03	25.02	22.52	22.10	23.15
hsa-miR-145-5p	24.82	26.63	25.41	22.89	23.16	22.62
bta-miR-99a-5p	25.98	27.71	26.63	25.26	25.63	25.95
hsa-miR-151a-5p	29.30	30.32	29.26	27.42	27.62	27.33
cfa-miR-101	29.84	30.76	28.57	26.82	27.46	26.86
hsa-miR-125b-5p	22.36	25.59	23.99	23.46	24.25	23.95
hsa-let-7b-5p	25.84	25.98	24.79	24.53	24.30	24.89
cfa-miR-23a	26.00	27.00	26.09	24.30	24.63	24.91
cfa-miR-30a	30.04	30.41	28.99	26.15	26.65	26.38
hsa-miR-19a-3p	27.33	27.78	27.70	26.11	25.69	25.56
hsa-miR-29b-3p	30.04	32.75	30.96	29.02	29.40	29.18
hsa-miR-7-5p	32.39	33.93	33.98	31.33	30.24	31.42
hsa-miR-362-5p	31.65	31.10	31.68	30.01	31.02	30.87
cfa-miR-1271	31.82	32.75	33.26	31.54	31.94	30.23
hsa-miR-93-5p	28.66	28.14	27.91	26.83	26.63	26.14
hsa-miR-214-3p	26.05	26.36	26.74	28.33	28.63	28.50
cfa-miR-181b	28.67	29.81	28.23	28.94	28.79	28.23
hsa-miR-27b-3p	25.78	27.01	26.59	24.88	25.13	25.22
hsa-miR-20a-5p	27.81	27.96	27.66	25.62	24.85	25.39
gga-miR-30c-5p	27.91	28.39	27.63	25.60	25.79	24.43

miRNA	Lung_56	Lung_57	OSA_58
hsa-miR-130a-3p	29.33	28.58	31.39

Biology Open • Supplementary information

hsa-miR-185-5p	30.96	28.81	29.36
hsa-378a-3p	30.22	29.09	27.24
cfa-miR-221	29.14	27.05	26.07
hsa-miR-22-3p	30.01	27.91	28.62
hsa-miR-16-5p	23.38	23.30	24.82
hsa-miR-145-5p	23.56	21.80	25.77
bta-miR-99a-5p	27.01	24.85	27.18
hsa-miR-151a-5p	29.23	26.55	29.45
cfa-miR-101	29.43	27.99	30.74
hsa-miR-125b-5p	23.52	23.24	25.33
hsa-let-7b-5p	24.32	24.09	26.54
cfa-miR-23a	24.39	24.32	26.47
cfa-miR-30a	27.69	26.85	30.47
hsa-miR-19a-3p	26.40	26.88	26.35
hsa-miR-29b-3p	31.24	28.97	31.62
hsa-miR-7-5p	31.18	29.17	29.58
hsa-miR-362-5p	32.12	29.44	30.45
cfa-miR-1271	30.83	30.88	32.97
hsa-miR-93-5p	26.79	26.93	27.55
hsa-miR-214-3p	28.21	28.47	26.27
cfa-miR-181b	29.37	27.21	30.20
hsa-miR-27b-3p	26.61	25.34	26.70
hsa-miR-20a-5p	26.62	25.85	25.75
gga-miR-30c-5p	26.47	24.81	27.41

Biology Open • Supplementary information

Table S6. Normalized Ct values for measured miRNAs in each cell line used for correlations with cell line doubling time.

	Cell Line			
	cOVC-OSA-31	cOVC-OSA-75	cOVC-OSA-78	cOVC-OSA-103
Mean doubling time (h)	17.51	22.47	27.24	52.07
miR-130a	1.45	3.68	1.7	2.54
miR-185	1.11	2.38	1.94	1.79
miR-378a	1.53	1.94	1.23	0.7
miR-221	-0.86	-0.32	-1.1	-2.74
miR-335	6.41	5.08	3.12	5
miR-22	0.37	2.08	0.11	0.67
miR-16	-1.61	-1.88	-1.72	-1.86
miR-145	4.9	-0.44	-1.93	-2.49
miR-99a	-2.17	0.86	0.28	-0.98
miR-452	6.41	7.43	6.83	4.49
miR-542	6.02	6.79	4.06	7.02
miR-9	5.02	3.35	3.02	7.02
miR-95	6.41	7.43	6.8	7.02
miR-223	6.41	7.43	6.8	7.02
miR-34a	6.41	4.81	5.61	1.72
miR-224	4.62	7.43	6.83	3.81
miR-182	4.45	1.81	2.29	7.02
miR-127	6.41	7.43	6.83	6.76
miR-151a	1.78	1.86	1.19	1.71
miR-708	3.35	6.45	6.27	7.02
miR-592	6.41	5.93	6.38	7.02
miR-218	2.59	7.43	6.62	6.72
miR-101	2.7	2.46	1.65	2.9
miR-133c	6.41	5.48	4.83	5.45
miR-128	5.78	6.62	5.83	6.87
miR-143	6.41	1.63	1.15	0.77
miR-125b	-4.28	-3.91	-4.4	-3.96
let-7b	-1.34	-1.69	-1.38	-2.32
miR-23a	-0.98	-3.14	-2.86	-2.51
miR-204	-0.54	4.92	4.79	7.02
miR-196a	4.6	2.84	2.83	4.69
miR-30a	1.21	1.73	1.05	2.76
miR-19a	-1.37	0.58	0.72	0.87

Biology Open • Supplementary information

miR-29b	3.35	4.41	2.85	2.78
miR-146b	3.22	7.43	6.83	3.99
miR-7	3.27	2.65	2.24	3.82
miR-1	6.41	7.36	5.32	6.9
miR-362	3.12	5.25	3.72	5.01
miR-1271	2.32	4.08	3.22	4.41
miR-93	-0.44	-0.18	0.18	0.61
miR-214	-2.7	-0.46	-0.76	-0.91
miR-183	5.3	2.7	3.16	7.02
miR-196b	0.73	3.49	1.53	2.88
miR-181b	2.41	1.69	1.4	5.43
miR-27b	-1.12	-0.4	-2.71	-1.92
miR-20a	-1.85	0.96	0.08	0.36
miR-30c	0.75	1.58	0.87	1.28
miR-96	6.41	6.56	6.03	7.02

Biology Open • Supplementary information
